# Supplementary material for: Purging due to self-fertilization does not prevent accumulation of expansion load
Source: PLoS Genet. 2023 Sep 1;19(9):e1010883. doi: 10.1371/journal.pgen.1010883 (PMC10501686; doi:10.1371/journal.pgen.1010883)
Supplement: S1 Table — Split times, in generations, for pairwise comparisons between populations from different regions. We calculated split times using dadi by fitting a bottlegrowth model with a population split. Comparisons between populations within the same region are omitted. (PDF) [file pgen.1010883.s003.pdf]

	Abruzzo				Apuan Alps					French Alps				Swiss Alps			
	Am	Gs	Gz	Mv	Ca	Gf	Po	Se	St	Br	Cc	Ga	La	Es	Ma	Pa	Pi
Am	-	-	-	-	1356	1380	1083	977	1077	1968	1447	1661	2013	1915	2047	1829	1997
Gs	-	-	-	-	1341	1307	1111	1001	1216	2134	1668	1900	2137	2063	2180	2014	2169
Gz	-	-	-	-	1336	1409	1058	1070	1002	1921	1395	1609	1979	1950	2083	1858	2031
Mv	-	-	-	-	1306	1299	1034	920	1057	2079	1496	1733	2117	1980	2157	1885	2089
Ca	1356	1341	1336	1306	-	-	-	-	-	2241	1947	2248	2427	2442	2580	2521	2595
Gf	1380	1307	1409	1299	-	-	-	-	-	2364	2079	2434	2505	2557	2669	2640	2691
Po	1083	1111	1058	1034	-	-	-	-	-	2275	1705	1997	2345	2320	2484	2262	2429
Se	977	1001	1070	920	-	-	-	-	-	2240	1746	2045	2295	2226	2343	2214	2363
St	1077	1216	1002	1057	-	-	-	-	-	2025	1550	1790	2161	2180	2315	2104	2253
Br	1968	2134	1921	2079	2241	2364	2275	2240	2025	-	-	-	-	520	447	1125	466
Cc	1447	1668	1395	1496	1947	2079	1705	1746	1550	-	-	-	-	506	524	935	538
Ga	1661	1900	1609	1733	2248	2434	1997	2045	1790	-	-	-	-	687	626	718	679
La	2013	2137	1979	2117	2427	2505	2345	2295	2161	-	-	-	-	547	402	881	441
Es	1915	2063	1950	1980	2442	2557	2320	2226	2180	520	506	687	547	-	-	-	-
Ma	2047	2180	2083	2157	2580	2669	2484	2343	2315	447	524	626	402	-	-	-	-
Pa	1829	2014	1858	1885	2521	2640	2262	2214	2104	1125	935	718	881	-	-	-	-
Pi	1997	2169	2031	2089	2595	2691	2429	2363	2253	466	538	679	441	-	-	-	-
