# Supplementary figures and images for: Purging due to self-fertilization does not prevent accumulation of expansion load
Source: PLoS Genet. 2023 Sep 1;19(9):e1010883. doi: 10.1371/journal.pgen.1010883 (PMC10501686; doi:10.1371/journal.pgen.1010883)

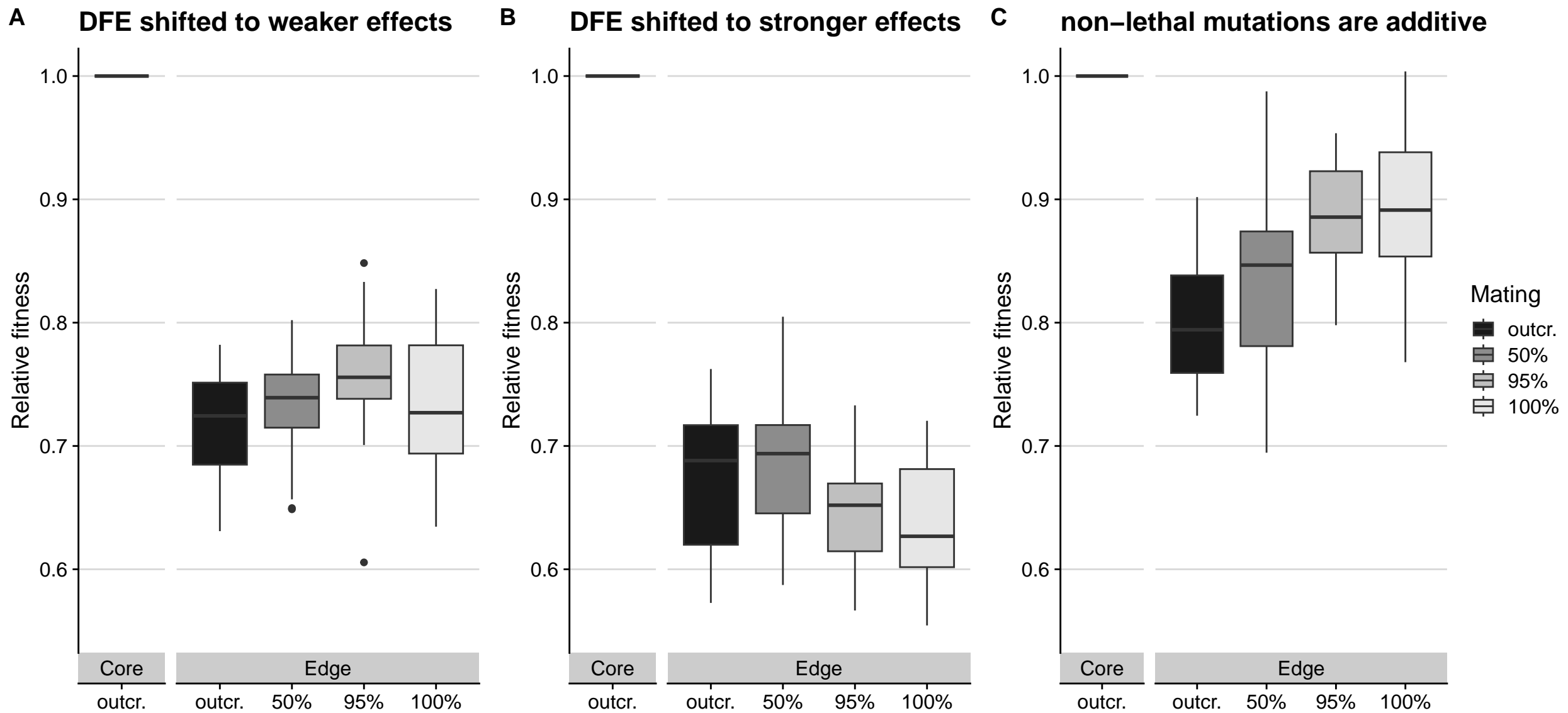

Supplement: S1 Fig — Similar to Fig 1D, we assessed relative fitness for additional simulations where non-lethal mutations where drawn from gamma distributions with with the same mean as our original parameter set (s¯=-0.001) but using shape parameters which shift the distribution to contain a higher proportion of weak-effect variants (α = 0.5) (A), or to contain a high proportion of large-effect variants (α = 2) (B). Lastly we also compared to a case using our original DFE shape (exponential distribution with s¯=-0.001) but instead with additive mutations (h = 0.5) for all non-lethal variants (C). Other parameters remained as described in the main text. (PDF) [file pgen.1010883.s004.pdf]

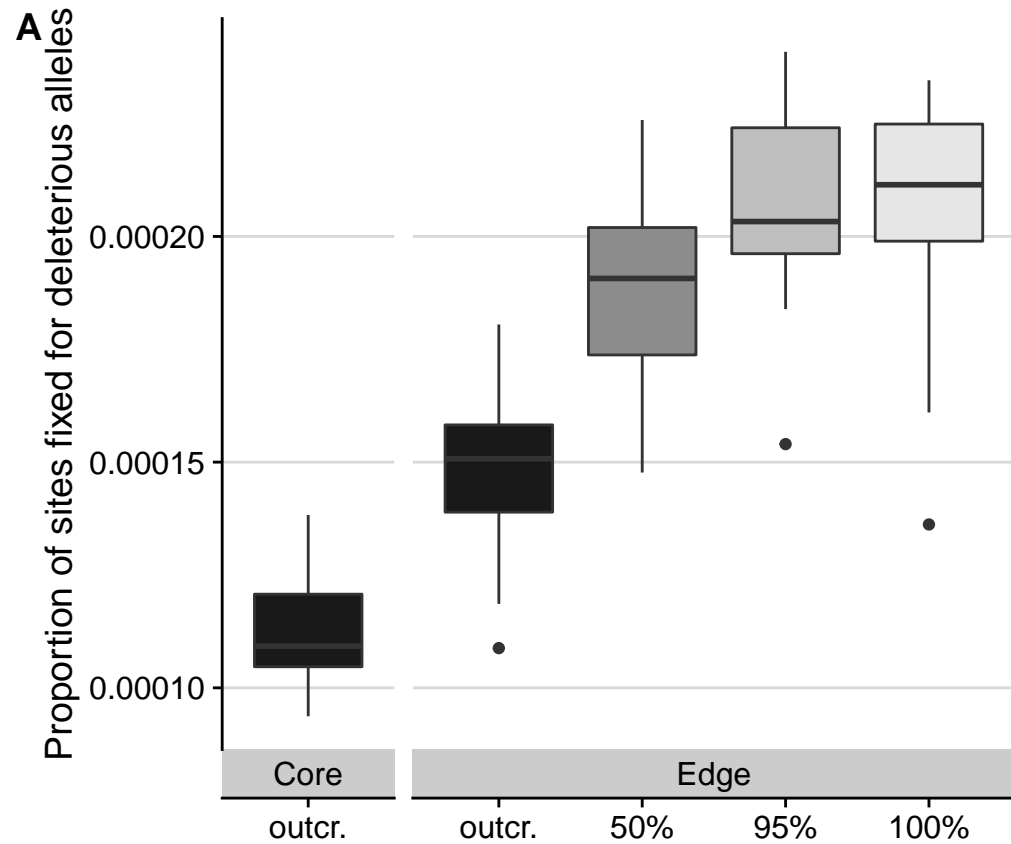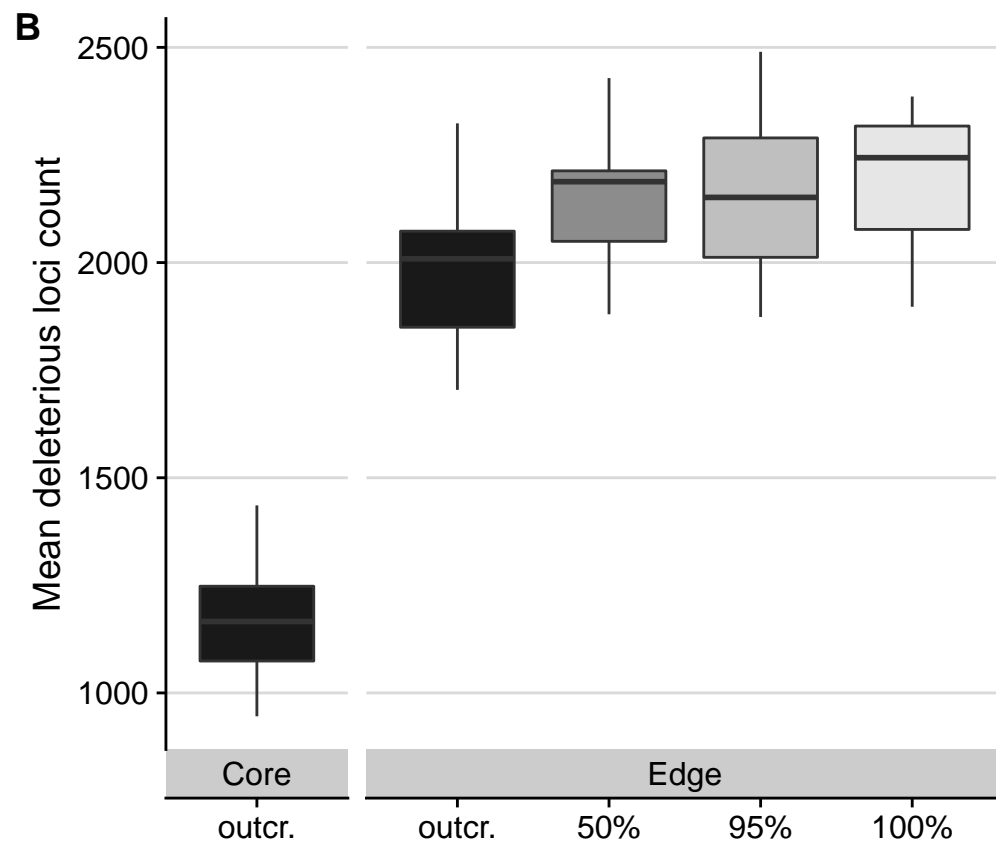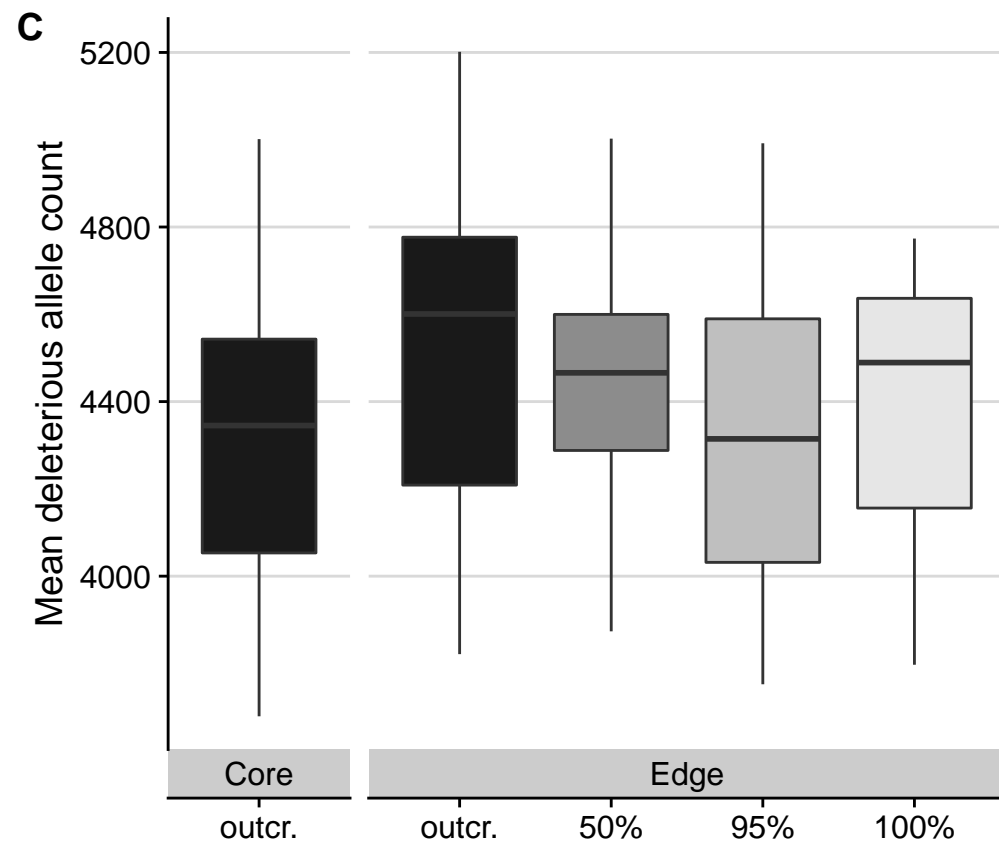

Supplement: S2 Fig — The proportion of sites fixed for deleterious alleles (A), the mean counts of deleterious loci (B), and the mean counts of deleterious alleles (C), all assessed at the end of the simulations for core and edge populations across selfing rates. Whiskers indicate the 1.5 interquartile range. (PDF) [file pgen.1010883.s005.pdf]

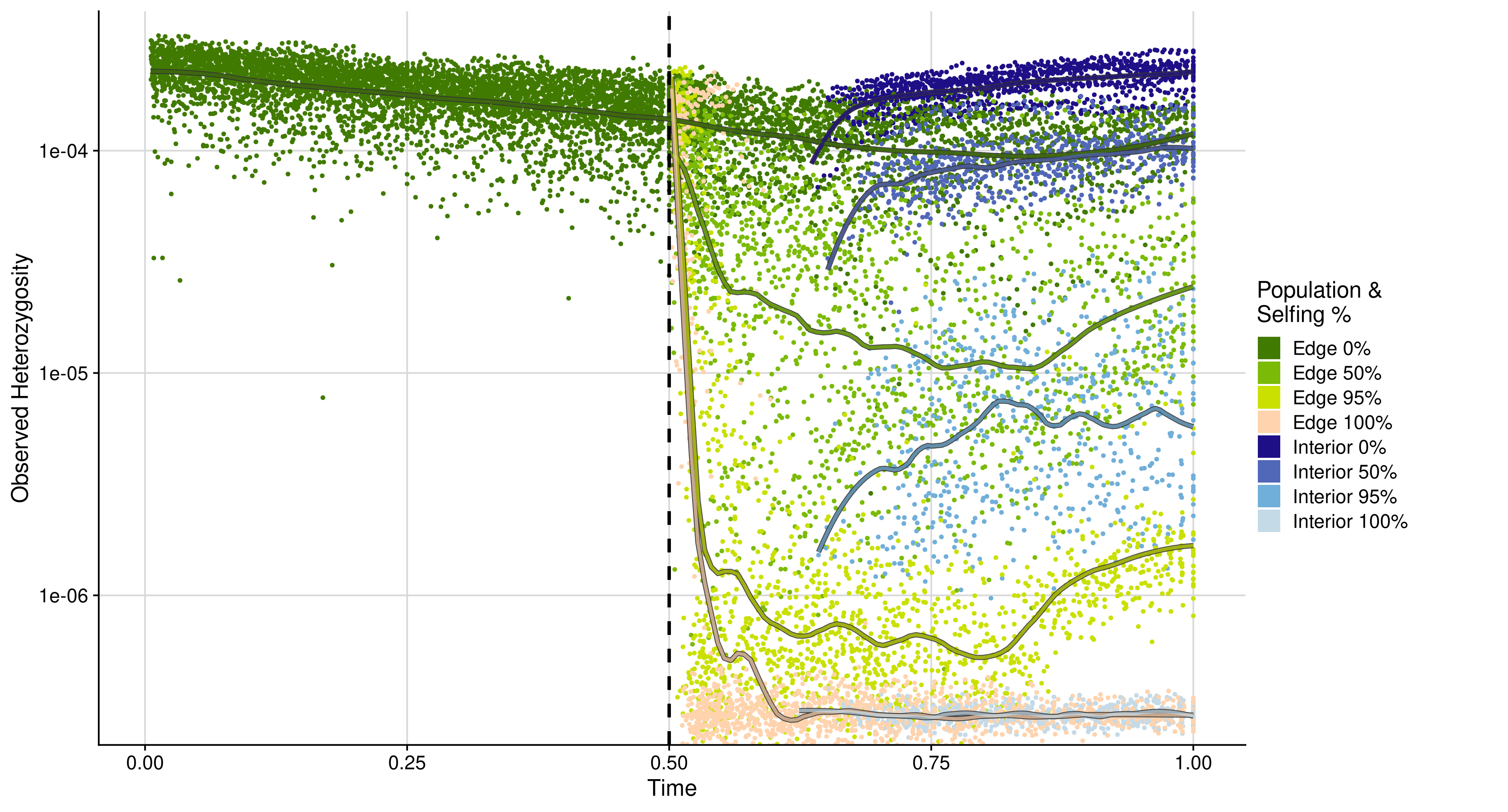

Supplement: S3 Fig — Trajectories for the mean observed heterozygosity over relative time, as described in Fig 2A, but now including all simulated selfing rates. (PNG) [file pgen.1010883.s006.png]

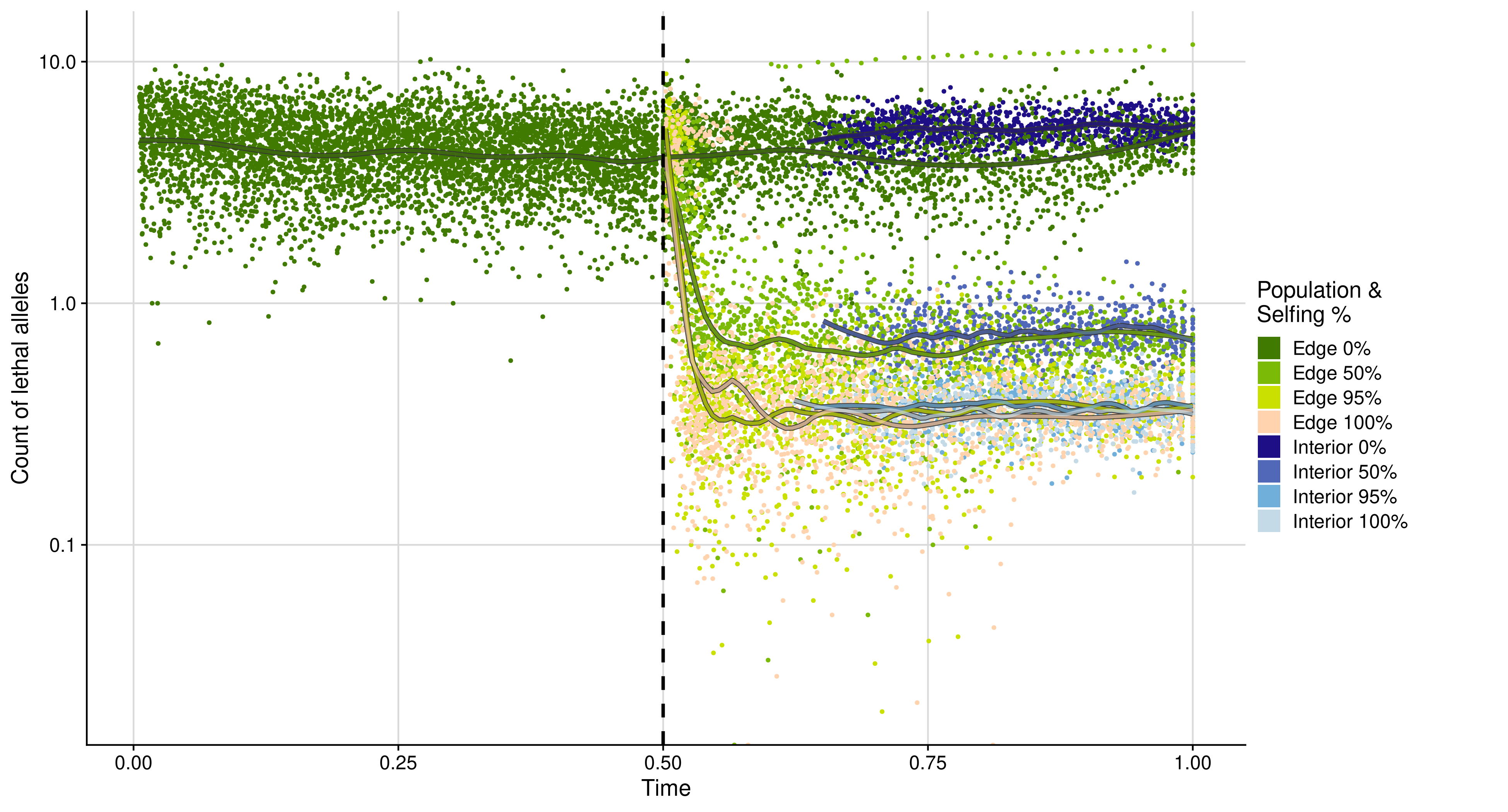

Supplement: S4 Fig — Trajectories for the mean count of lethal alleles over relative time, as described in Fig 2B, but now including all simulated selfing rates. (PNG) [file pgen.1010883.s007.png]

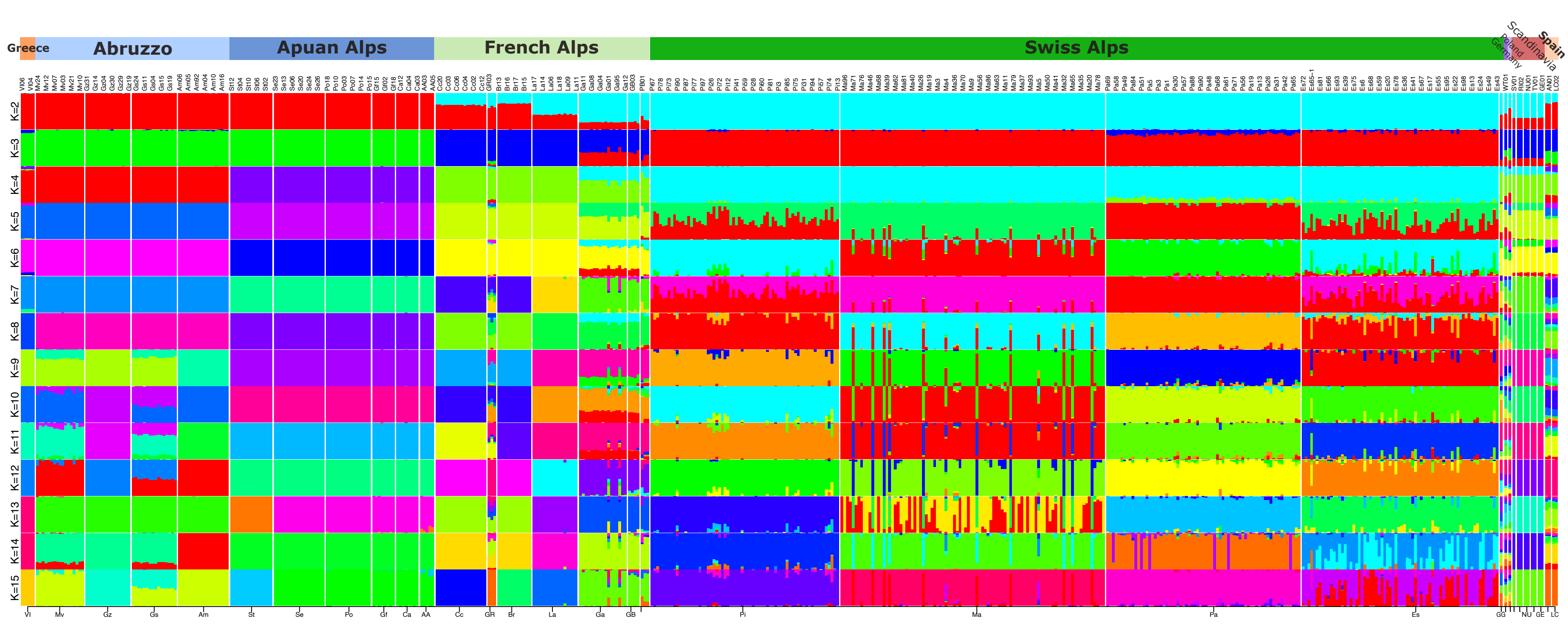

Supplement: S6 Fig — Results from K = 2 to K = 15 from admixture analyses run on the combined empirical dataset across Europe. The lowest CV error is for K = 14, however it is most useful to compare the populations structure across values of K to see how well this matches known geography and demographic history of the populations. We observe clean distinctions among our geographic regions sampled (indicated above the bar plots), with evidence for some gene flow across geographic space as one observes higher K values. (PDF) [file pgen.1010883.s009.pdf]

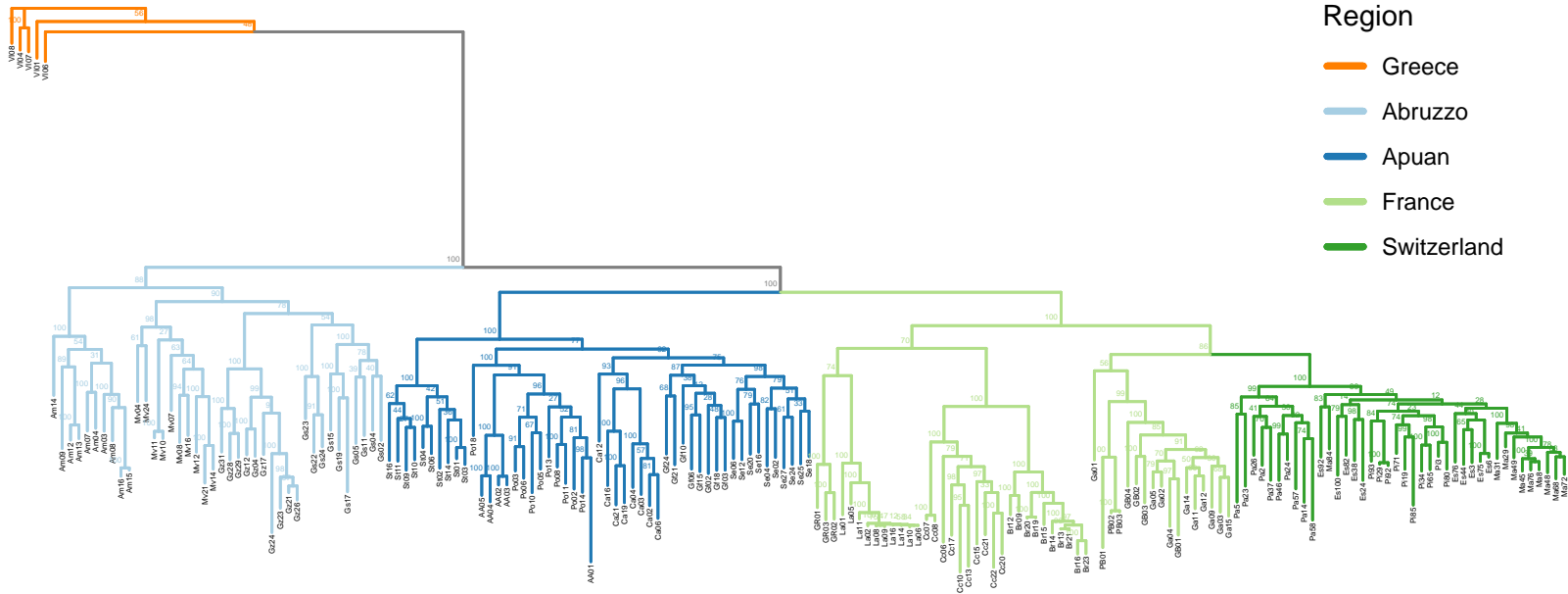

Supplement: S7 Fig — RAxML phylogenetic tree calculated using the rapid bootstrap analysis with 1000 replicates and search for bestscoring ML tree option in RAxML (option -f a). We used ‘GTRGAMMA’ as the substitution model, and A. alpina individuals from Greece (population ‘VI’) as outgroup. For computational reasons we randomly subsampled to a maximum of 10 individuals per population and used the same pruned SNP dataset as in the admixture analysis. (PDF) [file pgen.1010883.s010.pdf]

Abruzzo

Apuan Alps

French Alps

Swiss Alps

920-1409

1395-2137

1829-2180

1550-2505

2104-2691

402-1125

**Split times**  
between regions  
(generations)

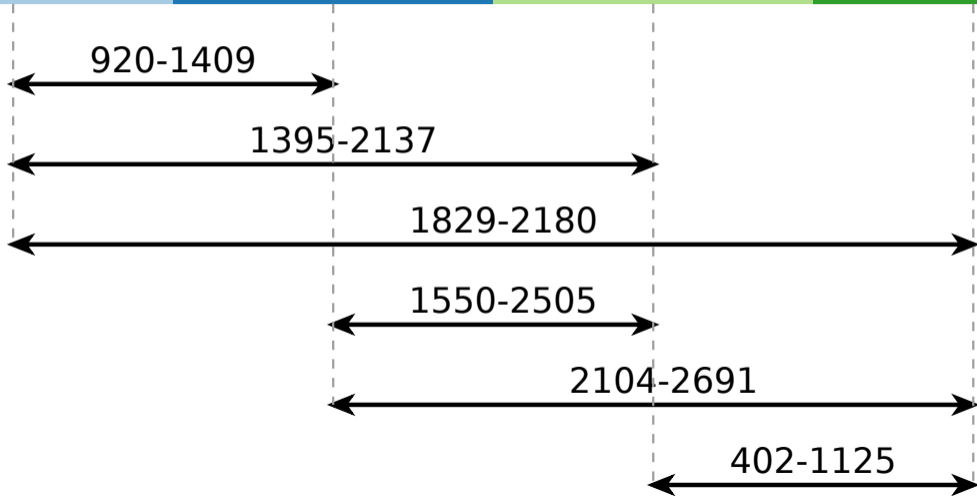

Supplement: S8 Fig — The range from minimum to maximum for split times between pairwise populations across regions is shown. Split times were estimated using dadi, and similar to the one-population demographic estimates (S9 Fig), we used 100 replicates and retained the best fitting replicate run based on log-likelihood. Estimates for every individual pairwise population across regions are listed in S1 Table. (PDF) [file pgen.1010883.s011.pdf]

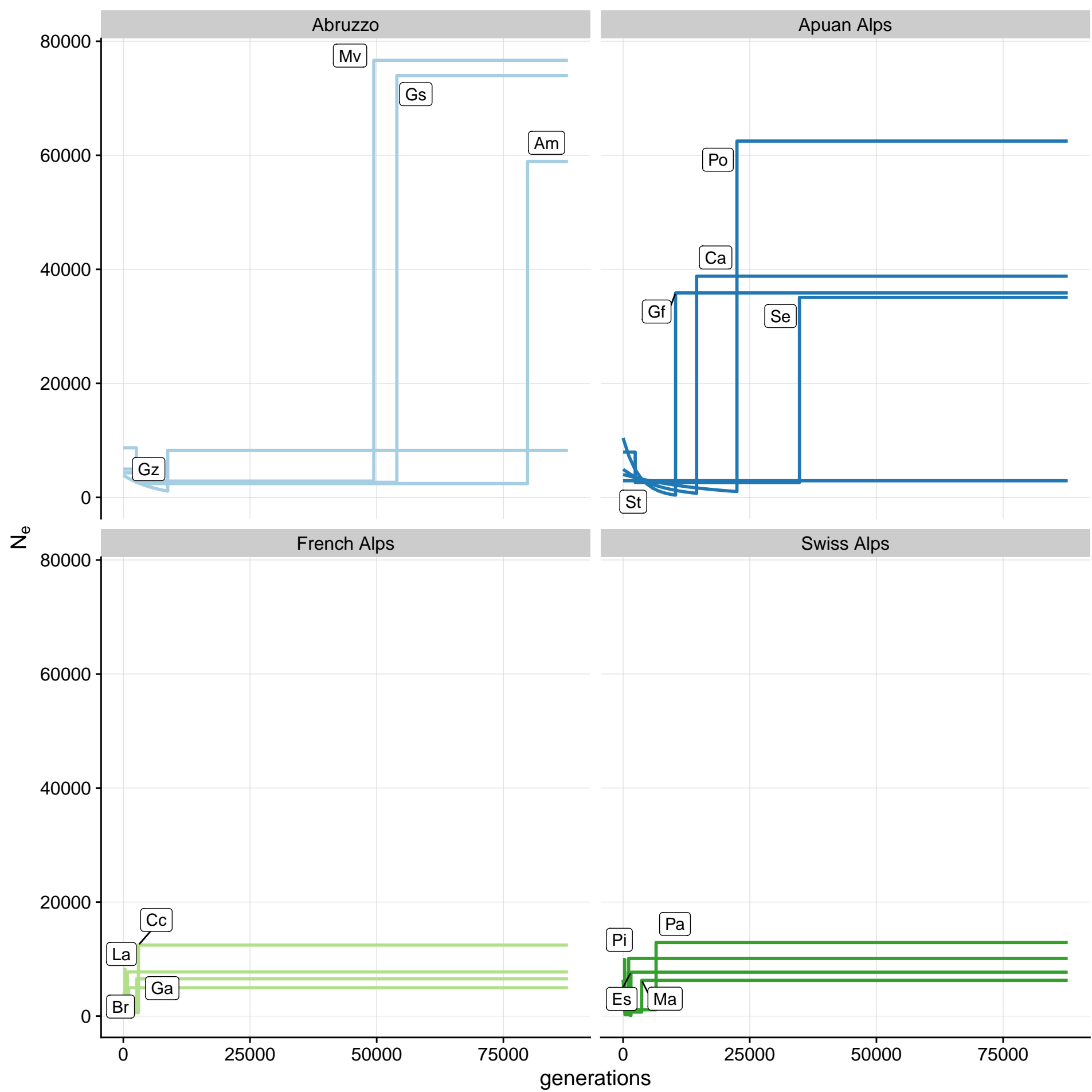

Supplement: S9 Fig — We inferred the demographic history of each of our newly sampled populations of A. alpina along with the densely sampled Swiss populations using dadi. This is a necessary step to account for the demography when inferring the DFE with fitdadi. This also allowed us to confirm if this newly inferred demographic history is consistent with past studies in A. alpina. The best-fitting models for our populations, based on AIC, were “bottlegrowth” models, indicating a past bottleneck followed by exponential growth (Es, Ca, Gf, Gz, Po), three epoch models, indicating a bottleneck followed by a sudden size change (Pa, Pi, Ma, Am, Br, Cc, Ga, Gs, La, Mv, Se), and the standard neutral model (St). Populations St and Gz were the only instances where competing models fitted approximately equally well (see S2 Data), therefore results for these population should be interpreted with caution. With the exception of Es, all Alpine populations best fit to three epoch models. Central Italian populations (light blue) show the most historic bottlenecks and the largest ancestral populations sizes. This is consistent with this region of highly outcrossing plants being subject to the last glacial maximum. Northern Italian populations (dark blue) show more recent bottlenecks and reduced ancestral sizes relative to central Italy, potentially reflecting their expansion northward. French and Swiss Alpine populations both showed the most recent bottlenecks and the smallest historic population sizes, consistent with both their shift to selfing and their more recent range expansion. Depleted genetic diversity along the axis of an expanding species range is expected, as is decreased Ne due to inbreeding and thus loss of diversity. These demographic inferences thus match our understanding of both the mating system shift and the range expansion that these populations experienced. (PDF) [file pgen.1010883.s012.pdf]

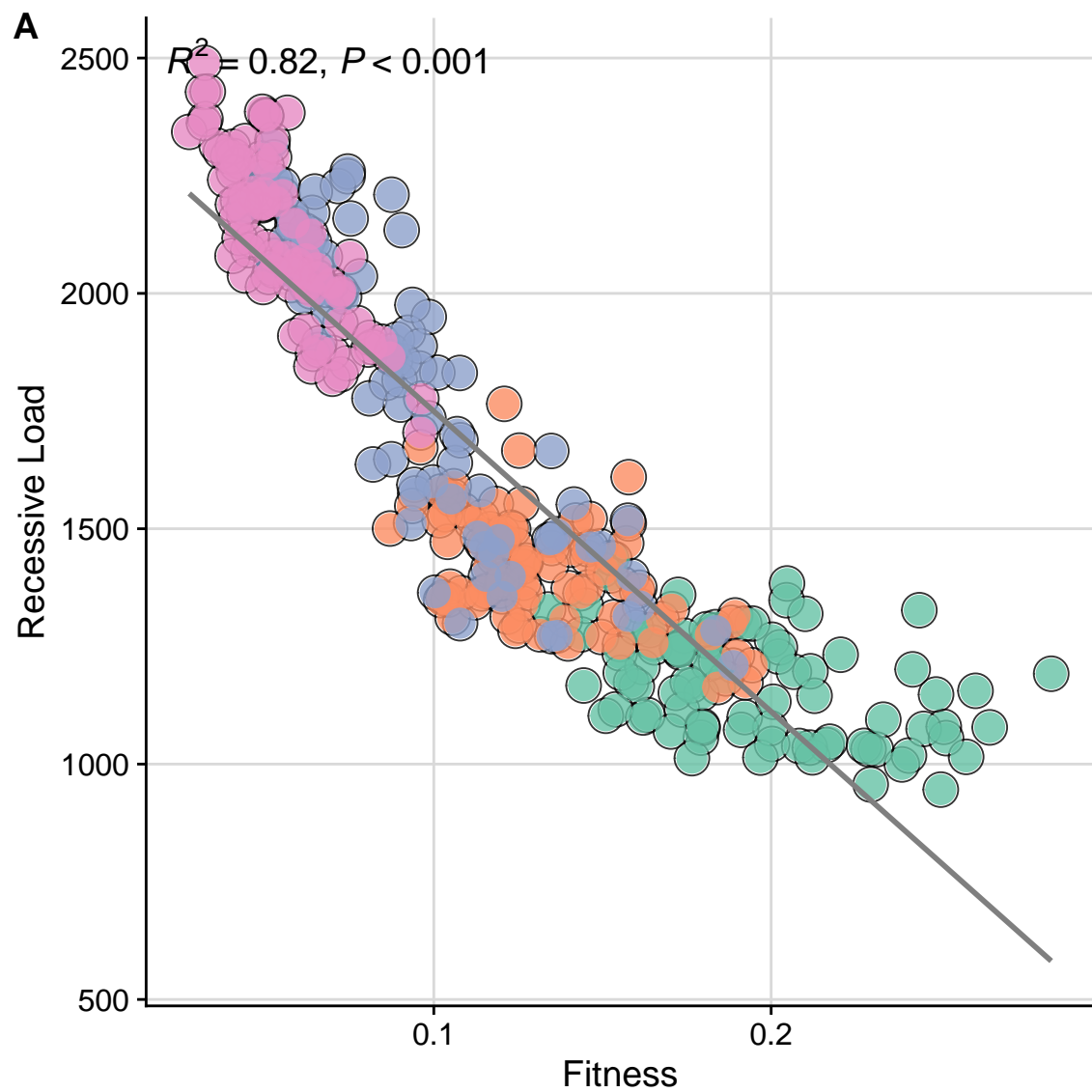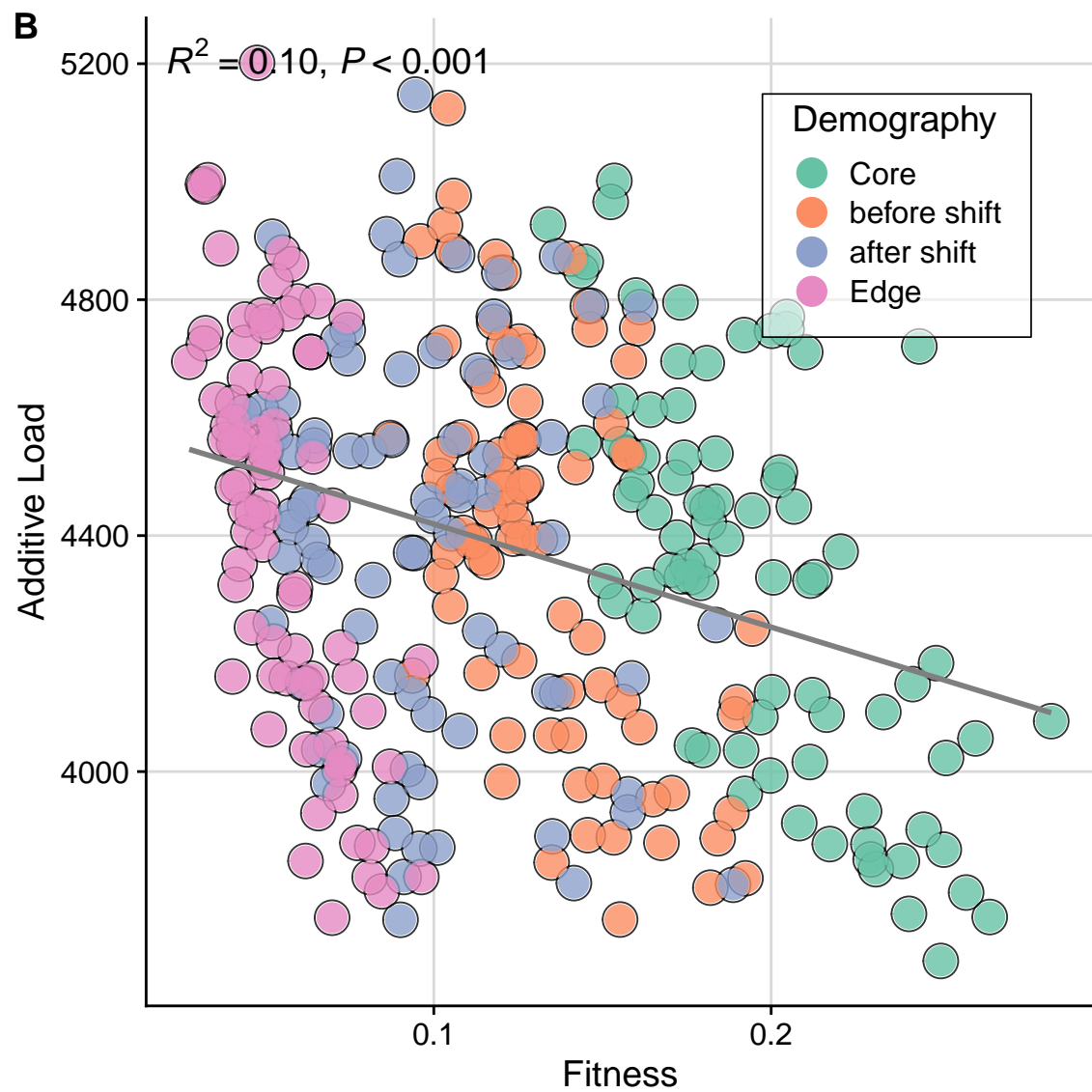

Supplement: S10 Fig — Observed (known) mean fitness from simulations for core (green), interior (orange, purple) and edge (pink) demes compared to the inverse of the count of deleterious loci (A), both after the range expansion is complete. The count of deleterious loci serves as a model for recessive load, which we find best correlates to fitness, compared to the additive model (B), where load is predicted by counting alleles. Results are for simulations with h = 0.3 for non-lethal deleterious mutations. (PDF) [file pgen.1010883.s013.pdf]

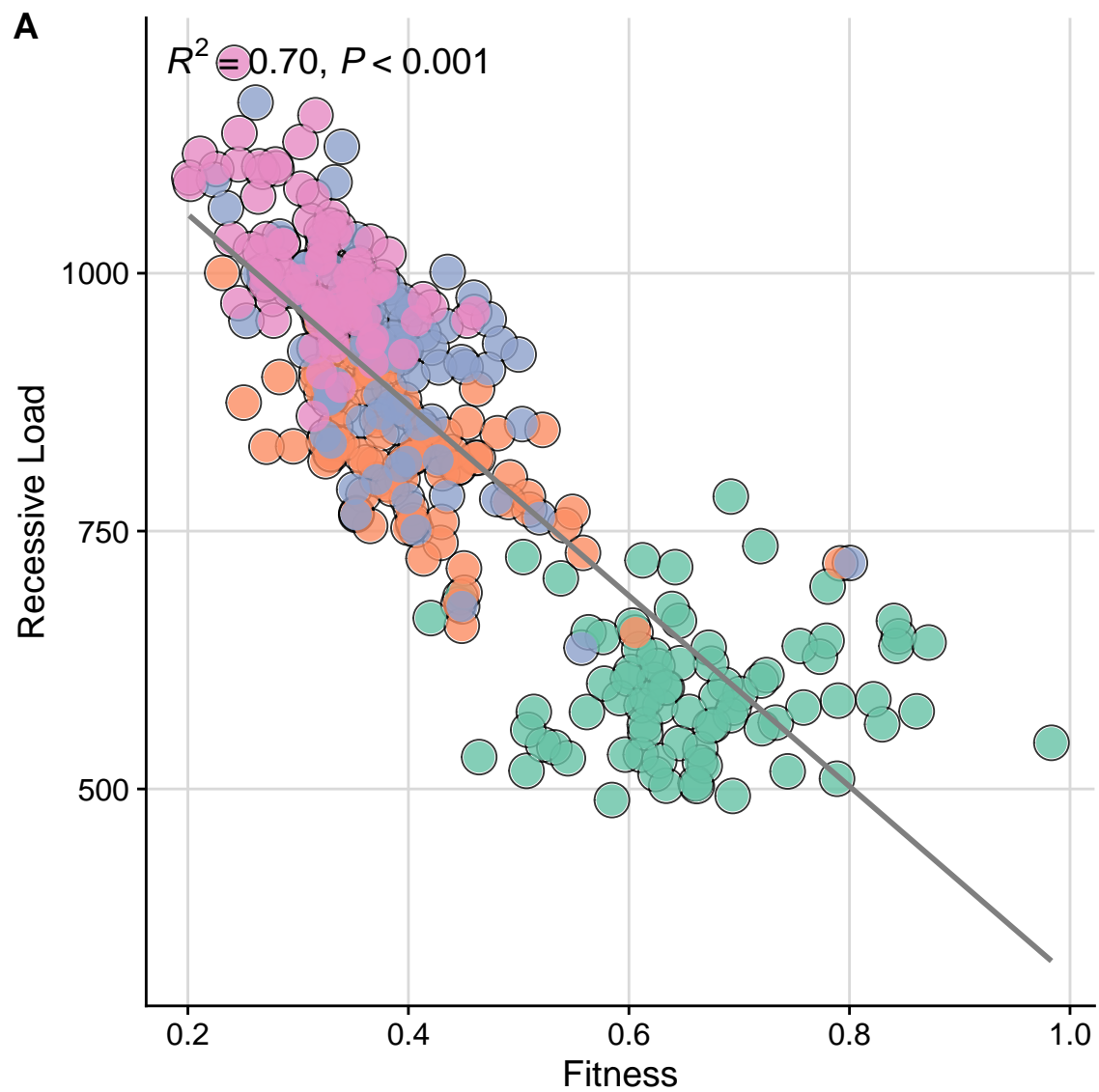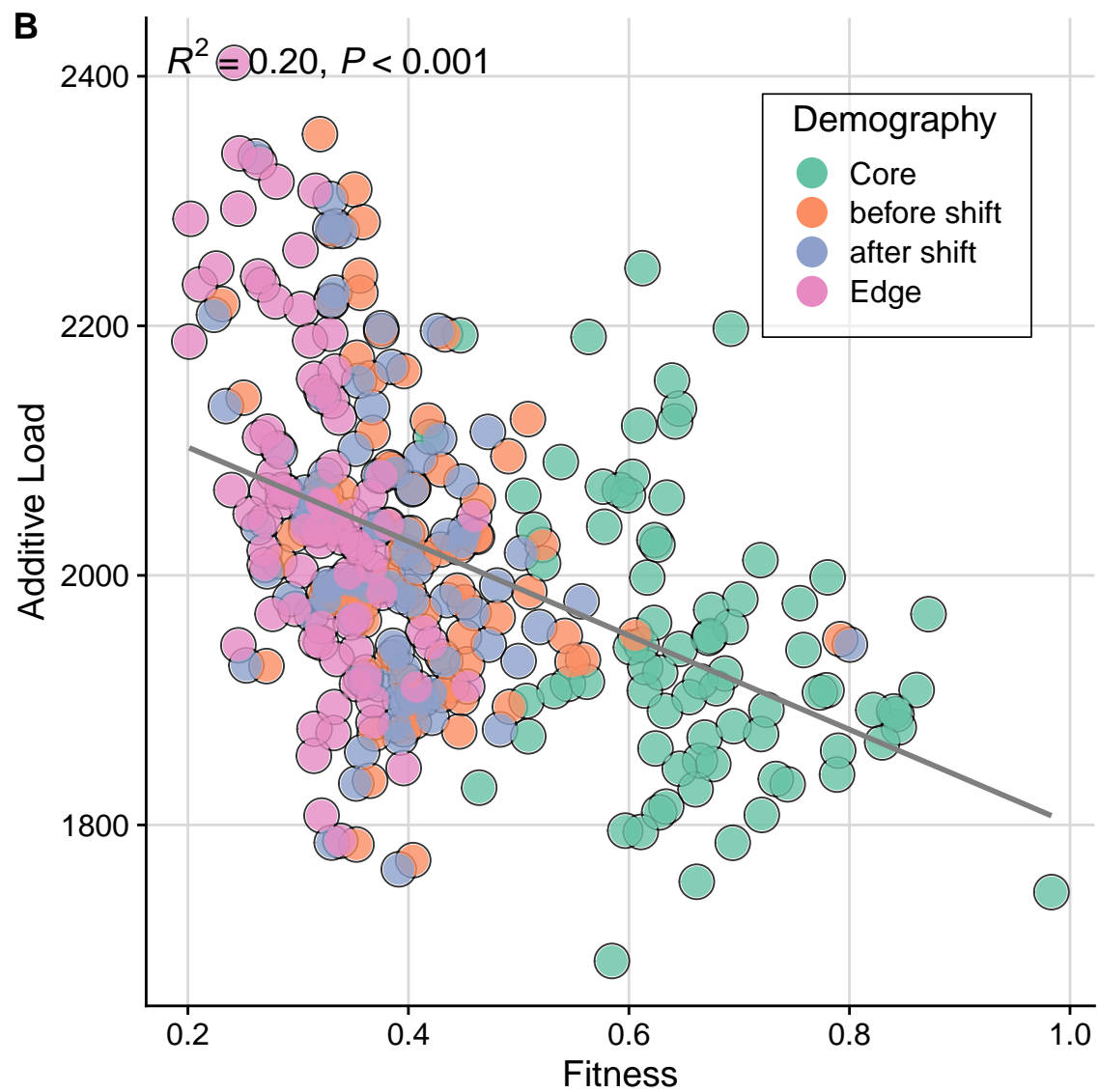

Supplement: S11 Fig — Recessive (A) and additive (B) genetic load compared with known simulated fitness to infer load when all non-lethal deleterious mutations are perfectly additive (h = 0.5). Data is from a supplementary set of simulations with these dominance parameters. This repeats the same analyses as S10 Fig, except now for simulations with additive mutations. This result again finds that the recessive model predicts load better (R2 = 0.70, P < 0.001) than the additive model (R2 = 0.20, P < 0.001). (PDF) [file pgen.1010883.s014.pdf]

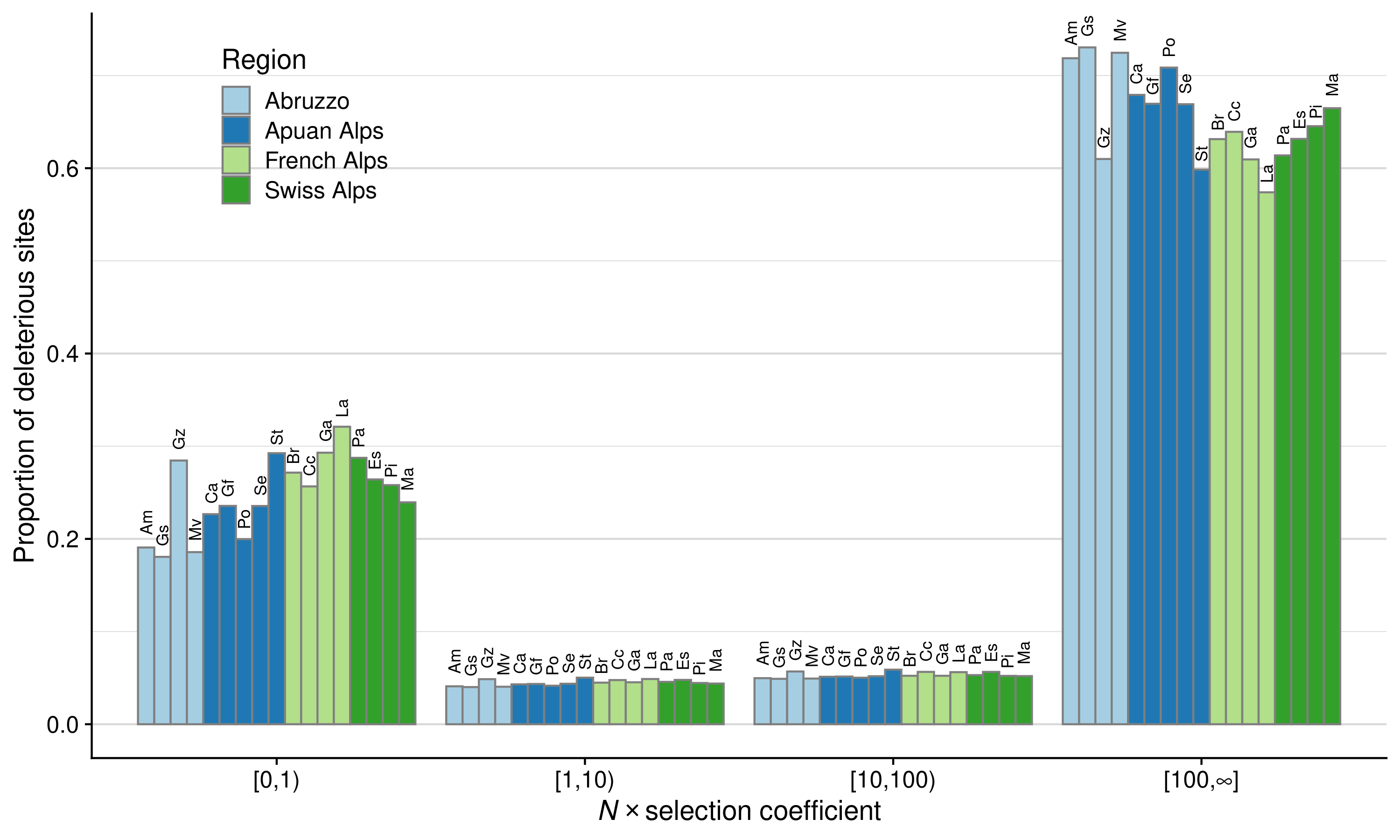

Supplement: S12 Fig — We inferred the DFE of each A. alpina population in the Italy-Alps expansion zone using fitdadi from dadi in python 3.8.12. We used the SNPeff annotation to construct polarized site-frequency spectra for neutral and deleterious sites after subsampling to a maximum population size of 20 individuals. To estimate demographic parameters, we tested the default single population demographic models (standard neutral model, two-epoch, growth, bottlegrowth, three-epoch) and two models accounting for inbreeding (standard neutral with inbreeding, two-epoch with inbreeding). We assumed a per base pair mutation rate of μ = 7 × 10^−9^ per generation, ran the default optimization for 100 replicates, and selected the best fit parameters within each demographic model based on likelihood and the best fit demographic model based on AIC. For fitdadi, we additionally assumed Lns/Ls = 2.85, dominance coeffient h = 0.3 and estimated the DFE for each model in 100 optimizations. We then chose the best-fit DFE optimization based on likelihood for each population for the previously chosen demographic model. DFE results from A. alpina populations across the Italian-Alpine range expansion for outcrossing populations from Abruzzo (light blue) and the Apuan Alps (dark blue) are compared to the selfing populations that have undergone range expansions into the French Alps (light green) and the Swiss Alps (dark green). We found mean proportions across all populations of 65.4% and 24.8% in the weakest and strongest selection classes, respectively. Less than 5% of sites segregated in the two intermediate selection classes. These proportions varied only marginally between core Italian populations (mean proportions 22.6% and 67.9% for weakest and strongest classes, respectively) and between expanded French and Swiss populations (means proportions 27.4% and 62.6% for weakest and strongest class). (PNG) [file pgen.1010883.s015.png]

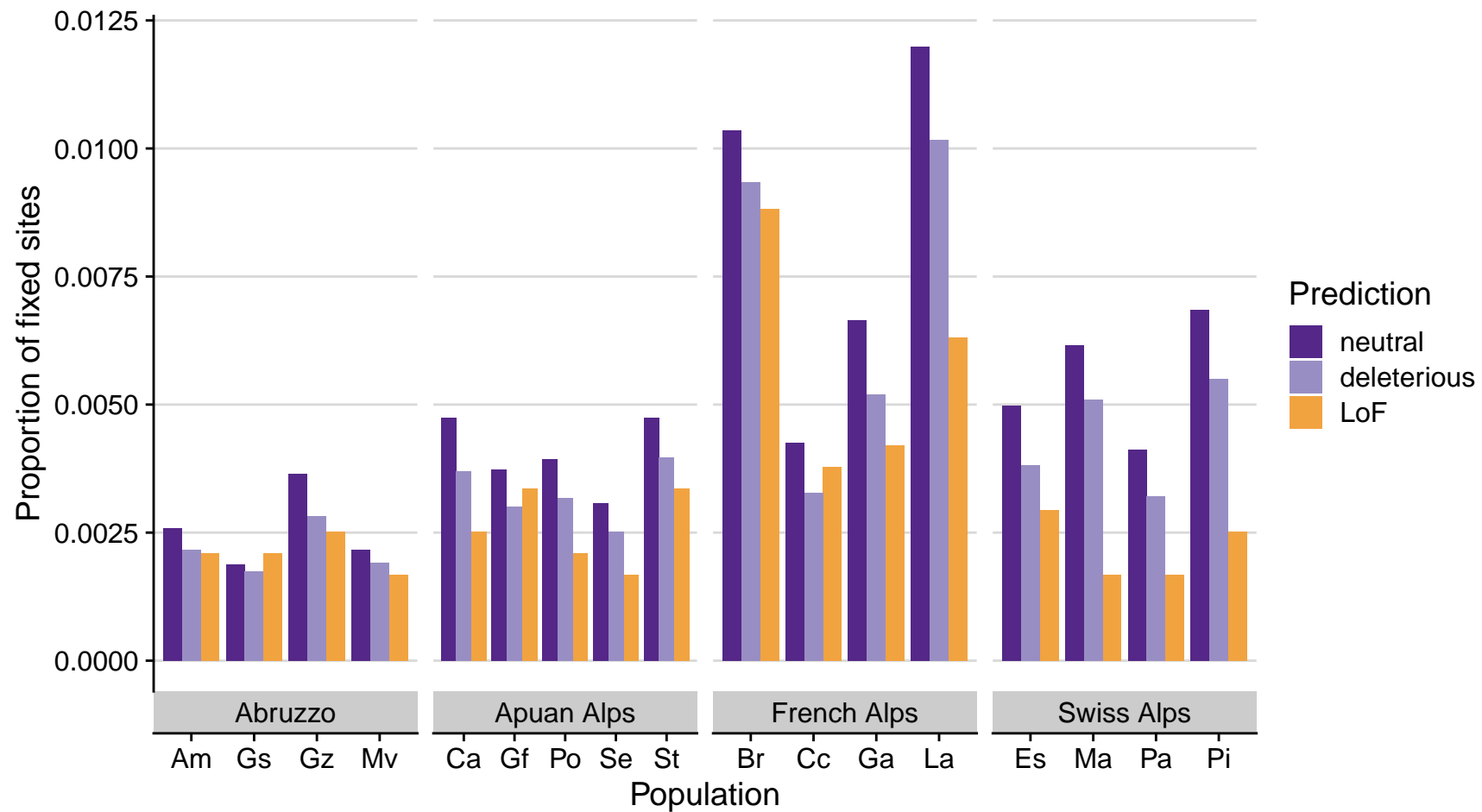

Supplement: S13 Fig — Fixation of predicted neutral (dark purple), deleterious (light purple) and loss of function (LoF, orange) sites per population. Y-axis shows the proportion of fixed sites in each focal population by allele category. We found that neutral sites fixed at the highest proportions (mean 0.505%), while LoF sites were at the smallest proportions fixed (mean 0.314%), indicative of their highly deleterious effect. French populations Br and La had the highest overall fixation proportions of any class (0.948%), while samples from the Abruzzo region had the lowest (0.228%). Swiss populations showed intermediate neutral fixation but LoF proportions similar to Italian populations. (PDF) [file pgen.1010883.s016.pdf]
