# Supplementary material for: Purging due to self-fertilization does not prevent accumulation of expansion load
Source: PLoS Genet. 2023 Sep 1;19(9):e1010883. doi: 10.1371/journal.pgen.1010883 (PMC10501686; doi:10.1371/journal.pgen.1010883)
Supplement: S5 Fig — Using the same parameter sets described in S1 Fig and similar to Fig 3, we compared the distribution of selection coefficients after the expansion. Insets emphasize strong reductions of proportions of lethal alleles with increased selfing, regardless of DFE or dominance coefficient parameterization. Error bars indicate 0.05 and 0.95-quantiles across the 20 simulation replicates within parameter combination. (PDF) [file pgen.1010883.s008.pdf]

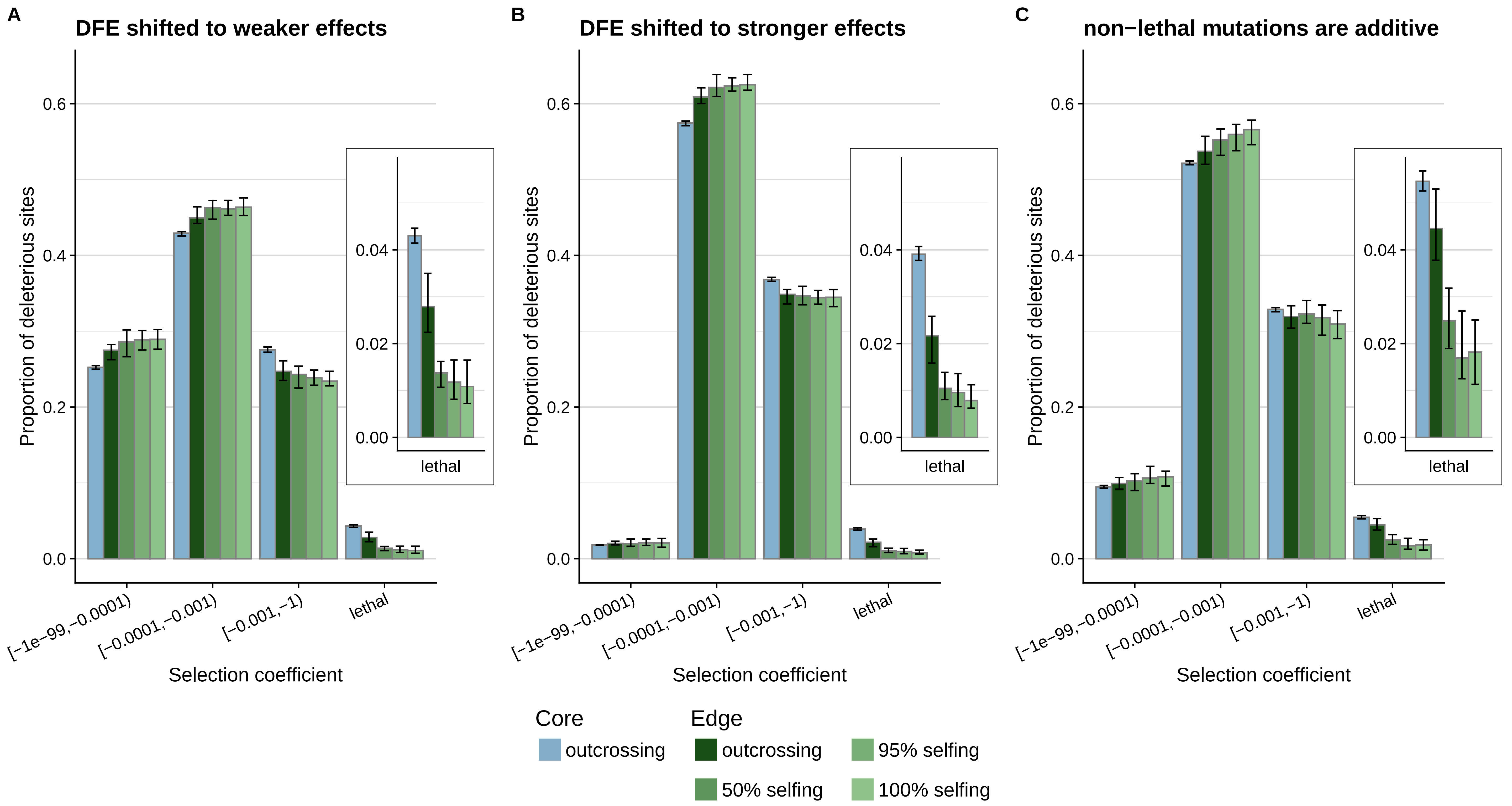
A
DFE shifted to weaker effects
B
DFE shifted to stronger effects
C
non−lethal mutations are additive
Proportion of deleterious sites
0.0
0.2
0.4
0.6
0.00
0.02
0.04
lethal
[−1e−99,−0.0001)
[−0.0001,−0.001)
[−0.001,−1)
lethal
Selection coefficient
Core
Edge
outcrossing
outcrossing
95% selfing
50% selfing
100% selfing
