# Supplementary material for: Purging due to self-fertilization does not prevent accumulation of expansion load
Source: PLoS Genet. 2023 Sep 1;19(9):e1010883. doi: 10.1371/journal.pgen.1010883 (PMC10501686; doi:10.1371/journal.pgen.1010883)
Supplement: Attachment — Submitted filename: RESUBMISSION_ResponseToReviewers.pdf [file pgen.1010883.s017.pdf]

**Dear Dr. Payseur and Dr. Mauricio,**

**Thank you for your handling of our manuscript; we have found the feedback of the three reviewers very useful for improving our study and appreciate the time they took to evaluate our work. Below in our replies, we specifically address each point from the reviewers in bold blue after each “>”. Line numbers correspond to the clean version of the resubmitted manuscript (not the track changes version).**

**Overall we have changed a great deal of the writing in the Introduction and the Discussion, addressed the relevant points in the Results and Methods, and added several new supplementary analyses. Additionally, here in our response to reviewers we also include some small analyses to reply to these points. We hope that this modified and improved version of our manuscript may be found acceptable for publication in *PLOS Genetics.***

**Sincerely,**

**Kimberly Gilbert**
**On behalf of all co-authors**

Dear Dr Gilbert,

Thank you very much for submitting your Research Article entitled 'Purging due to self-fertilization does not prevent accumulation of expansion load' to PLOS Genetics.

The manuscript was fully evaluated at the editorial level and by 3 independent peer reviewers. The reviewers appreciated the attention to an important problem, but raised some substantial concerns about the current manuscript. Based on the reviews, we will not be able to accept this version of the manuscript, but we would be willing to review a much-revised version. We cannot, of course, promise publication at that time and the manuscript will be sent to the same reviewers, but possibly different reviewers.

Should you decide to revise the manuscript for further consideration here, your revisions should address the specific points made by each reviewer. The main point of concern from the editor's perspective is the point made by reviewers 1 and 3 about the novelty of the study and being very explicit in setting up the questions in the introduction. The editors feel that the combination of the empirical work and the theoretical work is an asset. We will also require a detailed list of your responses to the review comments and a description of the changes you have made in the manuscript.

**Thank you for this feedback, we hope that the edits in our manuscript which we describe below in response to the reviewers have now better highlighted the novelty of our study and clarified the exact questions we address therein.**

Reviewer #1: Review of "Purging due to self-fertilization does not prevent the accumulation of the expansion load"

In this paper, the authors combine SLiMulations of expanding populations with differing mating systems, and the analysis of empirical population genomic data to investigate how mating system interacts with population expansion to determine the genetic load. I find some aspects of this work interesting, and the analyses largely strong, however, I have some trouble understanding the novelty /importance and situating this work in the broader literature. Below I outline this concern, and a few other suggestions which could improve the work.

On the whole I think that both the data and theory are promising, but neither was explored or presented in much detail and it is not clear they are sufficiently complimentary as to publish them together. If I was an author on this paper I would advocate for two papers at more modest journals - rather than packaging this all into one PLoS Genetics paper - but I am well aware that I am not an author :P

**> We thank the reviewer for this feedback, and though we can understand their point, given that our simulations allow us to thoroughly investigate and understand all processes acting and that the empirical work serves more as a potential case study with a more limited ability to fully understand the processes at play in nature, we intend to keep these findings as one overall study to investigate the combination of range expansion with mating system evolution.**

**We have re-written the majority of the Introduction and Discussion in light of this feedback, which we think has improved the clarity of goals and our findings' novelty and impact, fit them better into the field at large, and hopefully also granted a more detailed insight into each aspect of the overall study.**

** Context / Importance / Advance **
My greatest concern with the SLiMUlations revolved around the motivation for this work. We know that both selfing and range expansion reduce the efficacy of selection on the "average excess" and increase the exposure of deleterious recessive variants allowing for more efficient purging of highly deleterious receive variants. I believe the motivation of this work was to see how these forces interacted - but it was never fully clear to me what as at stake. The result that on balance on their SLiMulations these effects largely counteract one another such that there is no effect of mating system on the genetic load on balance is interesting, but is likely sensitive to the parameters chosen (e.g. a higher or lower proportion of highly recessive and highly deleterious variants could tip the scales). Stronger motivation up front about the outstanding question being addressed and why it matters more broadly (beyond "Whether this prediction holds when a species range expansion occurs concurrently with a mating system shift has, to our knowledge, not been fully explored") would strengthen this paper.

**> We thank the reviewer for the suggestions on altered selection and dominance coefficients. In the revised version of the manuscript, we included results from additional simulations where we explore the effects of shifted distributions of deleterious variants and further explore results from the simulations where deleterious mutations act additively. We find overall similar results as before but now further discuss these results (L. 173-176, L. 218-225, L. 357-360, Figures S1, S5). We**

**have also clarified our motivations in the Introduction (L. 79-82, L. 103-104) and laid out more clearly throughout the Introduction the relevant background versus unknown factors that we investigate in the interactions of selfing with range expansion.**

** A related concern was the connection between the theory and data **
The paper presents this empirical case as a difference in selfing vs outcrossing. However the data seem less clear cut. Assuming the "inbreeding coefficient," F , is F_IS, the extent of (?biparental?) inbreeding in the "outcrossing" populations seem quite high (nearly 50% applying the equation F = s/(2-s)), and the selfing rates in the selfer seem to be bout 90%. So, maybe our focus should be on those parameter values? Additionally, the bottleneck in simulation seems much more extreme (in terms of a reduction in sequence variation, than that studied in nature -- compare Figure 1C to 4C).

**> In general our simulations are not intended to match exactly to our empirical dataset, but instead serve as a model to inform the dynamics and processes contributing to realized load at the end of a range expansion. This is also why we simulated a varied range of selfing rates in our simulations.**

**Furthermore, given that none of our populations are expected to be at an evolutionary equilibrium, having undergone expansions and bottlenecks, the calculation of S from F_is would be confounded. Indeed the limited experimental studies available on outcrossing rates in the Apuan Alps identify 73-80% outcrossing (Tedder et al. 2011), which is potentially higher than the 50% that could be back-inferred by F_is due to non-equilibrium assumptions not being met in nature.**

**Therefore, while we agree with the reviewer that our study did not include assessment of outcrossing rates in *A. alpina,* in our revised manuscript review the literature in greater detail and added references for outcrossing rates in Italian and Alpine populations of *A. alpina* (L. 123). For a more extensive discussion of this aspect, see our reply to Reviewer 2's comment on our Methods section.**

**Tedder, A., Ansell, S.W., Lao, X., Vogel, J.C., and Mable, B.K. (2011). Sporophytic self-incompatibility genes and mating system variation in *Arabis alpina*. Annals of Botany *108*, 699–713. 10.1093/aob/mcr157.**

------ Technical concerns ------
In addition to these 'big picture' concerns I had some technical questions

** Use of the "Recessive load" calculation.**
The author's claim that the recessive rather tan the additive load calculation is more appropriate for nature populations because their simulations show that this is more strongly correlated with fitness (Figures S6 and S7). While there is indeed a higher R2 here, the recessive model seems to violate the assumptions of a correlation -- namely it appears that the residual value depends on its prediction and the prediction is generally quite poor for "core" sites. I'm not sure about the best way forward here, but while the R2 is clearly lower for the additive model, it seems unbiased.

> We have modified how we discuss this analysis in the main text (also in reply to reviewer 2). We agree that it is fair to say that the linear model of recessive load versus fitness seems to violate assumptions for a correlation. We do not, however, use this linear model to predict fitness, therefore we view the slight bias as non-fatal. We include this analysis and comparison in the manuscript merely to show the reader why we think both the recessive and additive models can serve as sufficient proxies that do both have some relationship relevant to fitness. We now clarified this in the main text in our revised version (L. 285-291, 381-384).

Below (Fig. 1 in this document), we provide some diagnostic plots on these linear models. Additionally, we log-transformed the values and refitted models, which did not improve these diagnostics (Fig. 2 in this document). We would also emphasize that the additive calculation (counting alleles, not loci) is not free of biases either, because of the low R^2, so neither is perfect for predicting fitness, but again we do not use these for prediction, only to assess whether there is any relation to fitness or not.

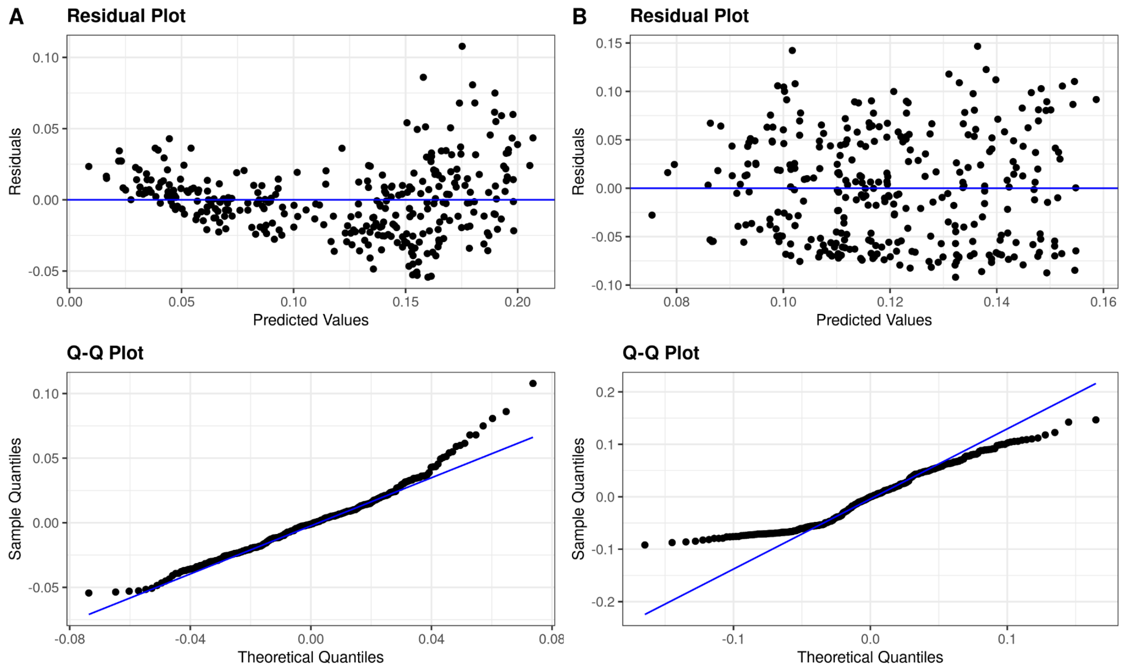


Fig. 1: Diagnostic plots for linear models described in the main text and depicted in the supplementary data, predicting fitness from (A) recessive or (B) additive proxy.

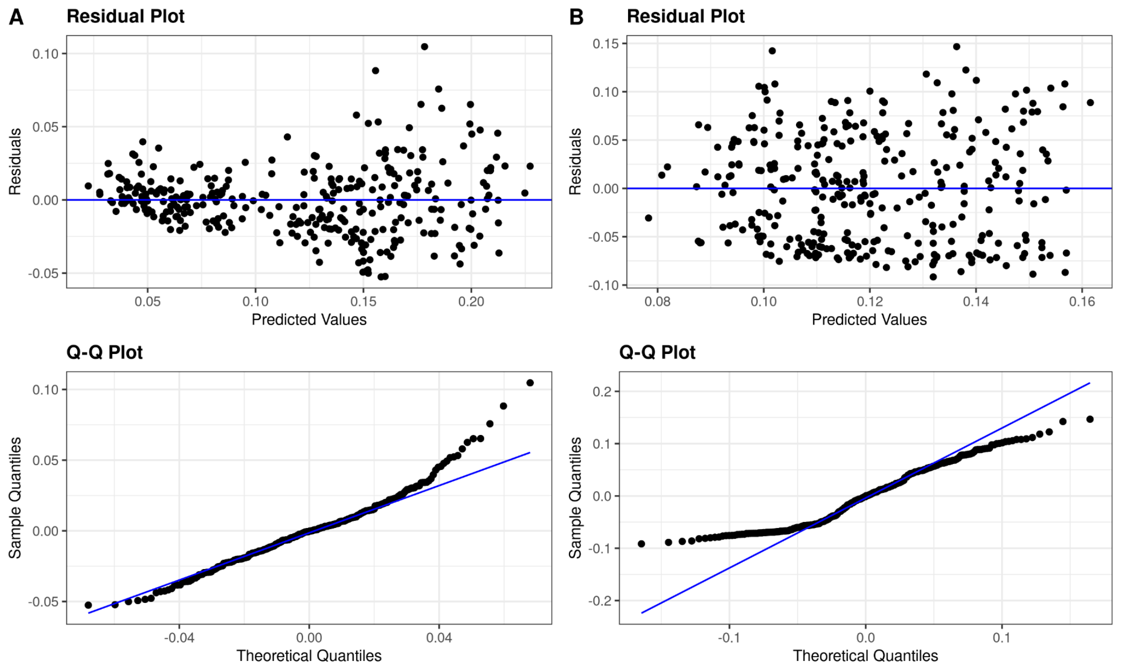


**Fig. 2: Diagnostic plots for linear models predicting log(load) from (A) recessive or (B) additive proxy.**

** Details of SLiMulation **
I had trouble understanding how selfing and mate limitation where baked into the SLiMulation. I know you can set a selfing rate in SLiM, but I also know that this selfing rate does not include "incidental selfing" in a randomly mating population. Did the authors add any mate limitation, if so what? If not, it seems that some incidental selfing occurred.. Anyways more details of this model would be necessary to evaluate it. Additionally, it is not clear if populations have a genetic selfing rate and it seems likely that selfing rates increase under mate limitation by e.g. geitonogamy, delayed selfing, and/or less competition between self and other pollen

UPDATE: Right before submitting this I saw the github link and found that the authors typed: initializeSLiMOptions(preventIncidentalSelfing=T), so that alleviates one concern.

**> As the reviewer themself later realized, we prevented incidental selfing for outcrossers so that we can be sure that all differences between simulation sets are due to the ability to self. We now directly mention this in the manuscript for extra clarity (L. 528-529, "obligately outcrossing"). The selfing rate is not genetic, but instead just set based on which deme an individual exists within so that we can be sure that 50% or 95% or 100% of individuals in a given deme are offspring of selfing events. We have now added a note in the manuscript about intentionally preventing selfing and further details about how selfing is set up in the simulations (L. 530-532).**

** Hazards of NGS approaches **
The authors combine different sort of data (i.e. depth sequencing technology etc etc differs,

see Supp1.csv), all of these issues, as well as divergence from the reference genome, can impact genotype calls, and could potentially introduce subtle biases into the analyses.

**> We thank the reviewer for this comment. We obtained published data from reviewed sources and combined it with high quality short read genomes. In addition to standard alignment, SNP calling and filtering procedures, we checked if depth is exceptionally low in any population (Fig. 3 in this document). While the Swiss populations have lower depth, they are still within acceptable range for short read genomes and populations from France show similar load and sequence diversity patterns. Depth doesn't seem to be associated with outcrossing rates. Our NGS pipeline follows what we consider to be standard approaches and cutoff thresholds. We did also realize the omission of one reference within this pipeline, now added in lines 593-594 for removing PCR duplicates.**

**Similarly, we checked if selfing or origin of the sample is associated with higher missing data in the genomes (Fig. 4 in this document), also in response to Reviewer 3. We find that population Ma has the highest maximum missing fraction, but still well below our cutoff of 20% described in the main text. We also excluded more individuals from the analyses due to low quality than previous published studies on the same data. Newly sampled French populations, which also have a history of expansion and selfing show similar patterns to the previously sampled Swiss populations. We are thus confident that our findings are not biased by low quality or insufficient filtering, but are happy to address this concern with the following figures.**

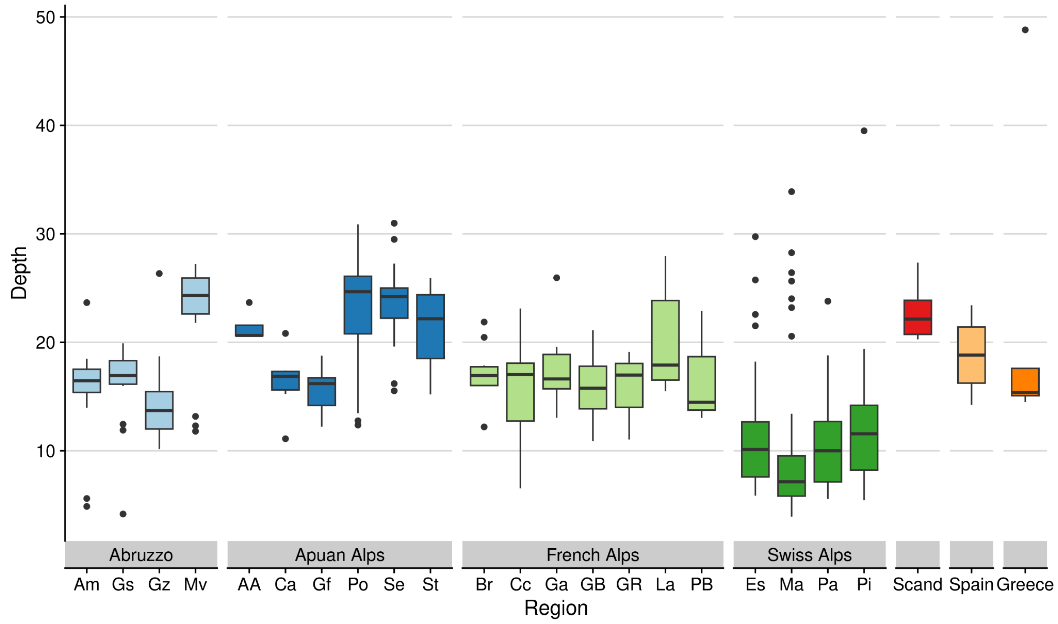


**Fig. 3: Distribution of depth in different populations**

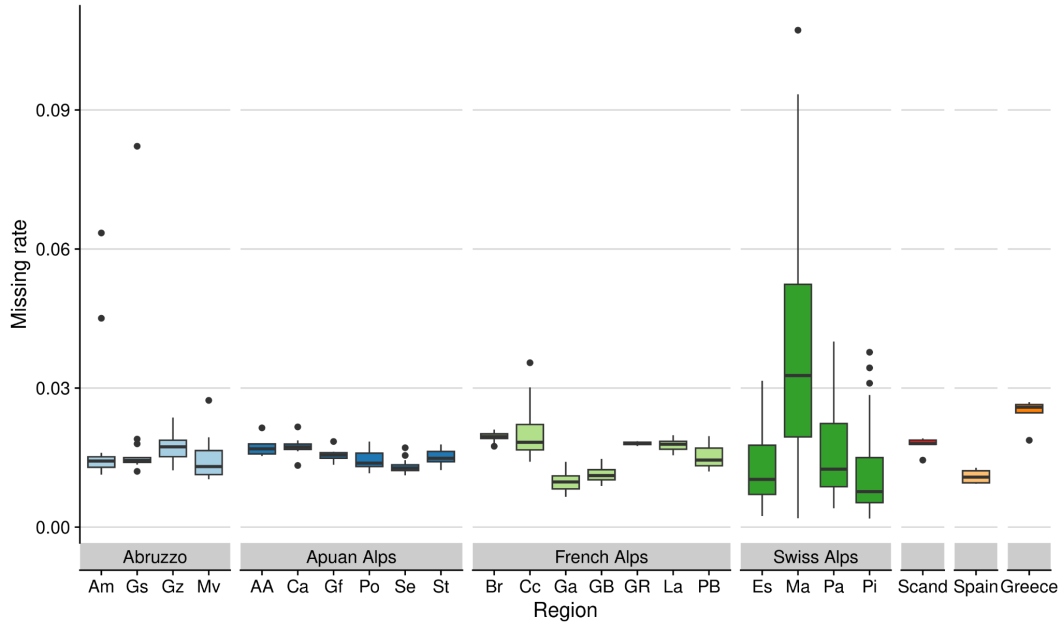


**Fig. 4: Distribution of missing data in different populations**

Reviewer #2: "Purging due to self-fertilization does not prevent accumulation of expansion load" (PGENETICS-D-23-00075)

In this paper, the authors conducted simulations to examine the relationship between different selfing rates and genetic load during range expansion using a stepping-stone model of migration to new demes. The authors compared core outcrossing demes to interior and edge demes that were either outcrossing, 50% selfing, 95% selfing, or 100% selfing. They compared the speed of colonization of new demes, nucleotide diversity, relative fitness, observed heterozygosity, the count of lethal alleles, the rate of fitness change over time, and the proportion of deleterious sites that fell within a range of selection coefficients from lethal to weakly deleterious. They found that selfers colonized demes more quickly, that nucleotide diversity, relative fitness, and observed heterozygosity were all reduced during range expansion and generally more for selfers than outcrossers, that the number of lethal alleles was greatly reduced for selfers compared to outcrossers, that the initial reduction in fitness was more dramatic for selfers compared to outcrossers, and that there was purging of lethal and somewhat deleterious alleles but that load of more weakly deleterious alleles did accumulate in selfers compared to outcrossers.

The authors then tested the hypothesis that they would see the same or similar results when comparing outcrossing and selfing populations of Arabis alpina. The selfing populations of this species are known to be the result of recent range expansion into the French and Swiss Alps from ancestral populations in Italy. They isolated DNA from 198 A. alpina individuals collected from selfing and outcrossing populations, used short-read sequencing, then assembled these short-read genomes and combined them with 342 existing short-read genomes for this species. They identified over 3 million SNPs in the 31 sampled populations,

then used this SNP data to calculate the inbreeding coefficient and nucleotide diversity in selfers vs. outcrossers, as well as use several measures to calculate if genetic load was accumulating or being purged in selfers vs. outcrossers. They found similar results in A. alpina compared to the simulations, including that alleles with large, deleterious effects (loss of function) were purged more in selfers but not weakly deleterious alleles.

Overall, I thought this was a very interesting study, and a nice pairing of theoretical and empirical results. The majority of the paper is very well-written and clear, as well as most of the figures. However, I have a few comments:

**> We thank the reviewer for their thorough reading of our manuscript and are pleased to hear that they found our study interesting, clear, and well-written!**

Figure 2, Figure S2, and Figure S3: I found the color contrast of these figures to be insufficient. I really had trouble distinguishing between the green and blue, especially the lighter shades. This was the most difficult on Figure S2 and S3, where there are finer gradations in shading of green and blue used for the different selfing rates. I can see the trends, but I can't see the difference between 50% selfing and 95% selfing, for example. I think more color contrast would improve the readability of these figures.

**> We changed the colors of Figures S3, S4 and 2, and log-transformed the y-axis in S3 for additional clarity.**

Methods section: What were the outcrossing rates of each of the 31 populations of A. alpina sampled for this study? I couldn't find that information anywhere and I would like to know how the outcrossing rates of these populations compare to what was used in the simulations. Are these populations closer to 50% selfing or 95% selfing?

**> We do not have information for every specific sampled population and we instead rely on the literature for knowledge of selfing rates across the European range of *A. alpina* (Ansell et al. 2008; Buehler et al. 2012; Tedder et al. 2011; Tedder et al. 2015; Laenen et al. 2018; L. 124-128). The inbreeding coefficients, nucleotide diversity and population relatedness analyses we show for our populations sampled across the whole European range support the reported view from the literature of selfing within alpine populations (as well as highly selfing in Scandinavia and Spain) and outcrossing within the Italian peninsula (and Greece). Tedder et al. (2011) is the only experimental source of estimated outcrossing rates, and they studied populations from the Apuan Alps, estimating 73-80% self-incompatibility, and one population from the central Alps, exhibiting 100% selfing.**

**As mentioned in our reply to Reviewer 1, we are also hesitant to back-calculate the outcrossing rate from the equation F=s/(2-s) since this calculation makes the assumption of an ideal population and given our demographic history of range expansion this assumption is certainly not met. Previous studies have inferred outcrossing rates in such a manner from genetic marker data and inferred "Outcrossing rates were considerably low ($t_{multilocus}$=0.315; $t_{singlelocus}$=0.118) indicating a high degree of selfing in *A. alpina*." for Swiss populations (Buehler et al. 2012) or "it**

**seems plausible that the Alps populations of *A. alpina* are diploid and at least partially self-fertile" (Ansell et al. 2008).**

**Overall, this would potentially place our expanded Swiss and French populations somewhere within the realm of 50-100% selfing, and our Apuan Alp populations as 20-27% selfing. Without direct estimations in the field of our specific populations, we think it is unwise to present or infer selfing rates more specifically within our empirical results.**

**Given that our simulations test a range of selfing rates and we do not see major qualitative differences among simulated selfing rates in terms of the impact on load accumulation, and because furthermore we can anyway not quantitatively compare values from our simulation results to values in our empirical results, we do not want to present outcrossing rates back-estimated from genomic diversity and represent a false level of confidence in the true outcrossing rates in nature. Furthermore, we do not have detailed direct knowledge of selfing rates among our replicate expanded populations within each region, and thus unfortunately cannot be precise about whether, for example, one of our Swiss populations that selfs more than another exhibits more or less load due to this difference in selfing rate.**

Results/Discussion: The authors state in the Results that there is more purging observed in the populations in the Swiss Alps compared to the French Alps. These are both regions with populations categorized as selfing. You can see these purging differences in Fig. 4D and Fig. S9, especially. But I couldn't find any mention in the Discussion of why the authors think they are seeing these purging differences. I would like to see that discussed explicitly.

**> We thank the reviewer for this valuable observation. We have now discussed this more extensively in the discussion (L. 388-392, in addition to clarifying the Results in lines 278 and 282-283). This could potentially be due to Swiss populations selfing to a greater extent than French populations and therefore more effectively purging deleterious and LoF mutations (in line with the trends suggested by our DFE analyses of higher selfing purging the most deleterious classes of mutations more effectively). Also see our answer to reviewer 3.**

Discussion, p. 11, lines 314-320: Here the authors discuss the recessive load model versus the additive load model and that there is evidence for load accumulation of one and purging of the other. The way this is discussed it sounds as if both models are true simultaneously and I am confused. The way I understood Figure 5, Figure S6, Figure S7, and what was stated in the corresponding section of the Results, was that the recessive load model was a much better fit to the simulation results, but that both models were applied to the A. alpina data and showed contrasting results. I think I am missing something here and I need clarification in the text (and perhaps in the Figure 5 legend) so that this makes sense.

**> Thank you for this comment. Along with our reply to reviewer 1, we have now clarified the interpretation of these two different dominance assumptions in the main text (L. 375-384) and in the figure 5 caption. We also rephrased the figure 5 caption for added clarity. Both models are correct to use, and provide different information and insight into our results, and the fit of each model to fitness as realized in the simulations is simply to substantiate that both models do have a relationship to**

**fitness under a history of range expansion.**

Discussion, p. 12, line 352: Currently says “Whether our sampled alpine populations populations”, but should say “Whether our sampled alpine populations”

**> Thank you for catching this typo, now fixed (L. 441-442).**

Discussion, p. 12, lines 363-365: The authors state here that they identified purging due to selfing but don’t know of other thorough investigations in empirical systems. I assume they are referring only to purging during a range expansion because they surely can’t mean purging in general. There are certainly other studies of purging due to selfing. The work of Michelle Dudash in Mimulus guttatus comes to mind. I do think that prior work on purging due to selfing should be compared here, even if it was not explicitly testing for purging during a range expansion. I think it would add to the quality of the Discussion to have a more thorough comparison to prior empirical work.

**> Thank you for pointing this out. Yes, we particularly meant evidence of purging during expansion and have clarified this, but this is a very good point of basing our results under the wider knowledge of purging in general. We have now added a mention of the work by Michelle Dudash and others in the Introduction (L. 86) and a small discussion of this in the Discussion (L. 429).**

Figure S9 legend: It reads “Y-axis shows the proportion of fixes sites in local population and allele category.” This sentence should read “The Y-axis shows the proportion of fixed sites in each local population by allele category.” Also, the last sentence says “Swiss population” where it should say “Swiss populations”.

**> Thank you again for spotting these mistakes, they have now all been corrected (now Figure S13).**

Reviewer #3: This is my review of the article “Purging due to self-fertilization does not prevent the accumulation of expansion load”. The study addresses the effect of range expansion with or without a shift to self-fertilization on the speed of colonization and the evolution of mutational load. The paper combines results of a simulation study with molecular data on a specific plant species.
I find the general topic of the manuscript novel and highly original. It adds to the relatively young field of range dynamics and mutational load with a very meaningful contribution. Of particular value are the results of the simulation study; the empirical results presented in the paper I found less convincing. The paper is generally well written, though the Introduction lacks the clarity; it could be better structured.

**> We thank the reviewer for their feedback and are glad to hear that they found the study to be a meaningful contribution that is novel and highly original! We hope that with our replies below and to the other reviewers that we have addressed the weaknesses mentioned in terms of clarity and structure in the Introduction (which has**

**been now rewritten) and for the strength of the empirical findings (to which we have added additional analyses).**

General comments:

1 Abstract and Introduction. Sentences that follow each other are sometimes disconnected (e.g., Abstract, sentences 1 and 2). Some terms that are used are too unspecific (e.g., "evolutionary challenges" in Abstract; L48 "difficulties at range fronts"; L62 "adaptive measures"). Both Abstract and Introduction would gain clarity if the potential effects of a mating system shift during range expansion were split into: the ecological advantages and – if any – disadvantages, and the evolutionary advantages and disadvantages. Right now, ecological and evolutionary implications are intermingled. As a result, it remains confusing what this study addresses and which outcome gives an answer to what. I would strongly emphasize this dichotomy of ecological and evolutionary implications, from Abstract to Introduction and later in presenting results and discussing them. E.g., the study of speed of colonization targets ecological aspects of selfing. Also, I strongly recommend parallel structure, always e.g., talking of ecology first and then evolution second, such that this separation becomes very clear.

**> We have now rewritten these parts of the Abstract (L. 12-16) to be specific about our meaning and to clarify the goals of our study. We have also rewritten the Introduction to improve clarity and the structure and flow within (L. 42 onward). This was very useful feedback for us, but in terms of the split between ecology and evolution that was suggested, it is one aspect we don't find that works for our study. Even though the ecological aspect – speed of expansion – is analyzed in our simulation results, we cannot comment on this at all for the empirical analyses since it is unknown how quickly *A. alpina* colonized the Alps. Our main focus is on the evolutionary side and load accumulation (speed of expansion is also an interesting result that we report but is not a main take-home from our findings), so in our rewriting we try to better emphasize this while still maintaining overall clarity and not excluding the fact that selfing does allow populations to colonize faster. We have added subsection headings also within the Discussion to further clarify the distinctions among our findings and their implications, and to separate the speed of expansion from our other results on load accumulation and purging.**

2 The simulation study is definitely the strong part of the paper. However, it is not really introduced in the Introduction; there, more emphasis is given to the empirical study system. I suggest to clearly state that simulations were done and what the goals were first, and then mention the empirical system and the goals of that one second. Introduce them both by providing similar levels of detail. What were the specific hypotheses in the two parts? Similarly, the Results section should be clearly split into two parts: outcome of simulations, outcome of empirical study. It would help to see two titles that reflect this split. Another split within those two parts should separate ecological implications of a mating system shift – speed of colonization, and evolutionary implications of a mating system shift – changes in load. Also, I recommend this split for the Discussion – simulations/empirical study + ecological/evolutionary implications. The discussion could emphasize more the novelty of the simulation results, and cite more empirical papers that have addressed mating system

shift in the context of range expansion and magnitude of load (novel papers of Siberian and North American Arabidopsis).

**> We thank the reviewer for this valuable feedback, particularly in highlighting our simulation results more in the Introduction as well as for the structuring throughout the remainder of the manuscript. We have now greatly revised the Introduction, also including more emphasis on our simulations, describing the goals and approaches used here and separating this from our introduction of the empirical system. We have also rewritten parts of the Discussion and related reworkings mentioned in our reply to reviewer 1. Within these revisions we have added more references to published works in the context of range expansions with selfing shifts and load accumulation.**

3 Simulations. Range expansion was modelled across a one-dimensional linear landscape. I could imagine that the magnitude and effect of drift may be different (reduced) if the landscape was two-dimensional. So far, most simulation work on expansion load was on 2-dimensional landscapes. I think that authors need to address potential deviations in one way or another – by verifying their results in 2-dimensional landscapes or by comparing their predictions with those e.g., produced by Peischl et al. under similar settings.

**> Thanks to the reviewer for pointing this out; that is true that 2-dimensional landscapes can lead to altered quantitative outcomes for load accumulation (essentially due to larger effective population sizes at the range front, but also to lateral gene flow) We have now added some discussion of how this may alter outcomes for our specific scenarios, given what has been found in other simulation studies with two dimensions (L. 450-455). We also point out that, as shown by Peischl et al 2013 (**https://doi.org/10.1111/mec.12524**), one-dimensional landscapes with *Ne* values such as ours should be good approximations for range expansions along narrow, two-dimensional corridors, which seems most fitting for our alpine plant species.**

4 I have a problem with the empirical part of the study. A first problem is a lack of information on the expansion history. The aspect of expansion is key to the research presented, and therefore, the authors need to provide data on how the expansion progressed in space in the study organism. The authors cite Tedder et al. 2015 which I checked. However, that study only showed that 3 outcrossing populations of Arabis alpina from central Italy and 3 selfing populations from the Alps fell into two separate clusters of microsatellite markers. This is no evidence that the species colonized the Alps from refugia in central Italy. The authors provide more structure results with their data, but those do not provide any insights into the past expansion history either. To learn about that, authors would need to produce some rooted population relatedness tree. Alternatively, they need to present results on demographic modelling in the main paper and provide data on split times among Italian populations and populations of the Alps that match those of glacial retreat.

**> The reviewer makes a valid point and made us realize one error that helps partly alleviate this issue. The citation to Tedder et al. 2015 was the wrong study and we have fixed this now to "Tedder et al. 2011", which is the appropriate reference for the mating system shift during expansion. Additionally, we added another reference for**

**the expansion history and refugial theory in alpine *A. alpina*, Ehrich et al 2007, who additionally studied the relatedness of *A. alpina* across a larger part of its range. It is important to note here with the reference to Ehrich et al. that there are expected to be multiple sources for the expansion into different parts of the Alps, and our samples from the Western Alps, for example, have a likely different origin from populations in more Central or Eastern Alps. We do not consider any of those more eastern alpine populations and to further elucidate the exact origins of our alpine populations, performed additional analyses as suggested by the reviewer.**

**As an aside, given that our new genomic dataset provides much improved power within the Italian peninsula to compare to our samples in the western Alps, we are glad that the reviewer requested this additional analysis and think it greatly strengthens our investigation of expansion load across this geographic transect and provides some novel insights into the history and relationship of these populations.**

**Our initial Admixture results in a small way support the expansion from Italy into the Western Alps, for example when K=2 in figure S6, there is a clear cline in relatedness within the French Alps, where French populations are intermediate between Italian populations and Swiss populations. At the next K=3, we see a clear split of the Italian populations and then a hint of relatedness between some French populations and the Swiss populations.**

**To improve upon this, as suggested by the reviewer, we analyzed split times using dadi, pairwise between populations in each of these regions and also thoroughly investigated relatedness among our populations by estimating a phylogenetic tree with RAxML. Both of these analyses are now included in the main text and discussed in lines 240-258 and supplementary figures S7 and S8. To also here briefly describe these results, we find clear concordance with an expansion originating in the Italian peninsula and colonizing into the western Alps. Using the Greek population as an outgroup, we observe that the Abruzzo samples in central Italy clearly exist as a monophyletic clade, and then the clade that contains the Apuan Alps and the French and Swiss Alps share a common ancestor separate from Abruzzo. Within that Apuan/French/Swiss clade, then the French and Swiss Alps share a more recent common ancestor than with the Apuan Alps which have their own monophyletic clade. Demographic analyses to infer split times also match these results (Figure S8, Table S1), showing recent splits between France-Switzerland, between Abruzzo-Apuan Alps, and then between Apuan-French Alps.**

5 The second problem is the estimate of load used in the paper. The authors introduce it very briefly such that it remains unclear what it really is. Also, I think it is not used often, and therefore, authors need to add other estimates that have been used e.g., in human pop. genomics or other lit. From what I read in the paper, the estimate is based on differences between populations in the count of derived alleles. Such an estimate gives a lot of weight to the many rare heterozygote variants that may contribute little to load if deleterious alleles are predominantly recessive (see Discussion in Henn et al. 2016 PNAS). I also do not understand what I should see in Fig 4D. First, the authors do not mention what each symbol

stands for. Then, I see that the purple dots have similar positions in the first 5 groups but are lower in the last group; among alpine populations, the difference in counts of derived, deleterious alleles was lower than the difference in counts of neutral alleles. Based on that, authors seem to argue for a history of purging. For strongly deleterious alleles, loss of function alleles, they find that differences are regularly lower in comparisons excluding Italy-Italy, but sometimes also higher. This suggests mixed evidence of purging for highly deleterious mutations. If my interpretation is correct, this would be somewhat against the predictions based on the simulations, wouldn't it? – All in all, this measure of load may be fine, but it should be – for reasons of comparisons and in line with the discussion in Henn et al. – be accompanied by other estimates of load (e.g., that give less emphasis on rare alleles that are mainly in the heterozygous state).

**> We estimate load through several different approaches in our manuscript, and it is not entirely clear to which of these the reviewer is referring to here, as they mention several different aspects of our load analyses, so we will address all. We have now added additional clarification in the main text in reply to this and other reviewers about what the additive and recessive load models are as well as clarifying the SNPeff and R'xy analyses. Here in the following text we will briefly explain these changes and point to the relevant lines in the revised manuscript.**

**Firstly, we annotated SNPs using SNPeff. SNPeff is frequently used in plant (e.g., https://doi.org/10.1038/s41467-022-35368-1) and animal genomics (see https://doi.org/10.1038/s41576-022-00448-x for a review), and has been cited 8335 times according to Google Scholar. Briefly, SNPeff classifies mutational impact based on functional prediction into different categories of functional change in coding regions for SNPs and small indels. This is described in L. 616-621). As with other methods such as phyloP, GERP, SIFT or PROVEAN, SNPeff has its limits and cannot infer s and h accurately, but for non-model organisms it is a commonly used method that makes use of functional information that is available without needing to construct a large multi-species phylogeny, required for conservation-based methods.**

**Using these annotated SNPs (synonymous, non-synonymous (missense and nonsense), loss-of-function) we then calculated R'xy. In line 269, the caption of Figure 4, and line 387 we add additional descriptions of which SNPeff classes go into which sets of R'xy analyses. R'xy was first introduced in human genetics and is an appropriate statistic because it largely avoids reference bias and can thus be used to compare different populations (see https://www.nature.com/articles/s41576-022-00448-x). We describe R'xy and its canculation and interpretation in L. 624-627 and L. 268-274. In the revised manuscript, we changed Figure 4D for clarity by changing the point type to better distinguish each estimate and removing the small confidence intervals that are smaller than the point size and thus confusing and distracting and not necessary. The colors are explained in the figure legend and in the caption. We have added more details about the interpretation of Figure 4D in the discussion (L. 385-398), and see also our answer to Reviewer 2.**

**We additionally use counts of putatively deleterious loci to calculate load under a recessive and under an additive dominance model. This is a common approach used**

**across human genomics and non-model organisms, and theory has well-predicted the expectations for how these load models behave during range expansions, e.g. Peischl & Excoffier 2015. We calculated additive and recessive load, just as it is often done in human genetics (e.g., https://www.nature.com/articles/nrg3931#Sec2, also discussed in Simons and Sella 2016) as well as also in plant and animal genomics (e.g., https://www.nature.com/articles/s41477-020-00834-5). Detailed descriptions of the additive and recessive load model calculations and meanings which can be found in the simulations section of the manuscript, since that is where we first use these estimators (L. 569-573). The way we calculate load is similar to the reference given by the reviewer (Henn et al. 2016), except we do not assign selection coefficients to mutations, because we do not use GERP scores and rather use all sites classified by SNPeff as deleterious. The reviewer also states a concern about rare heterozygote variants contributing to a greater signal of load while not actually significantly impacting fitness since most deleterious mutations are expected to be recessive. This concern would only be valid if, first, we relied solely on the additive model and, second, if that additive model were the only result to show an increase in load. First, we do not solely rely on the additive model; we also use a recessive model of load which exactly addresses this point by counting homozygous deleterious sites - see Figure 5A. Second, the additive model does not show an increase in load (Figure 5B), so any additional weight from rare heteroyzgotes only make our purging result more conservative. In response also to reviewers 1 and 2 we have added clairty in the main text to these descriptions and interpretations (L. 285-291, 375-384)**

**Lastly, we also use the DFE, estimated using fitdadi, to characterize the newly entering deleterious mutations, which is also often done to analyze the fate of load-contributing mutations (e.g., see Laenen et al., 2018). The methods for this are described in the caption of figure S12.**

6 A third problem is the fraction of missing data across sites/variants and the fraction of missing data per individual, especially if the latter is geographically biased because of differences in coverage. Cutoffs for missing data were set very liberally. --- Missing data may be biased towards regions of the genome with higher mutation rates that also do not align well. While this may mainly increase variance in results, any geographic pattern in bias would become problematic. I recommend being more stringent, which would result in still high enough SNP numbers (now >3 Mio.).

**> We have double-checked the filters that we ran on missingness (also in reply to Reviewer 1, please see above), as we did have the same initial worry as the reviewer for such potential biases. We show this result above in reply to Reviewer 1, that there are no major biases by region and we thus think that our filtering was sufficiently stringent to avoid major biases across geographic regions.**

Specific comments:
L48-50. The authors seem to have plants in mind and mention pollination. But what about other Allee effects affecting other types of organisms? Else, mention that in plants, pollination is vulnerable to an Allee effect.
**> In our rewrite, we now early on make it clear that this study will focus on plants (given the mating system shift being the major factor we investigate with range**

**expansion). We have also modified this sentence about Allee effects to clarify that pollination is subject to Allee effects (L. 63-64).**

L42-62. The paragraph is not as clearly written as it could be. I recommend writing in a more structured, more condensed way, mentioning the evolutionary implications that range expansions have, and then raising the theme how a shift in mating system may change predictions, and that this is what was addressed in the article.
**> Thanks for this comment, given this and other feedback we have largely reworked and rewritten the entire Introduction to better structure both the background and the goals of our study.**

L63-71. This paragraph (and the next) would benefit from a clearer structure, introducing the potential ecological advantages of selfing, and its evolutionary advantages/disadvantages, in the context of range expansion. I would first introduce the relevant theory and then the empirical results found so far. Or, in other words, I would e.g.., devote separate paragraphs to the theme of range expansion and mating system shift to selfing driven by selection for reproductive assurance.
**> See our reply to the previous comment, again useful feedback which led to a large rewrite of the whole Introduction.**

L72-. An Allee effect is based on ecology. Low density or small population size lowers fitness (positive density dependence).
**> That is correct, we have reworded this sentence (along with much of the Introduction) to clarify that as a result of the Allee effects, there is limited mate/pollinator availability.**

L77-80. Reference missing.
**> We added additional references for the consequences of self-fertilization:**

**Pollak, E. (1987). On the Theory of Partially Inbreeding Finite Populations. I. Partial Selfing. Genetics *117*, 353–360. 10.1093/genetics/117.2.353.**

**Charlesworth, D., and Wright, S.I. (2001). Breeding systems and genome evolution. Current Opinion in Genetics & Development *11*, 685–690. 10.1016/S0959-437X(00)00254-9.**

**Nordborg, M. (2000). Linkage disequilibrium, gene trees and selfing: an ancestral recombination graph with partial self-fertilization. Genetics *154*, 923–929.**

**In the revised version this section is in L. 95.**

L159-162. What is the difference between mean counts of deleterious alleles and counts of deleterious alleles? Why the difference in outcome?
**> The difference between the two counts is the first are counts of homozygous**

**deleterious *loci,* the second are counts of deleterious *alleles.* The difference in the outcome can be explained is due to the underlying assumption of the dominance coefficient.**

**We reworded the section (L. 178-181, L. 627-630) to clarify and avoid future misapprehension. Interpretations for the difference between the two models are discussed from L. 374.**

Methods. Was there a difference in average coverage (after filtering) for selfing populations?
**> There was no difference in selfers versus outcrossers (see our response to reviewer 1 above).**

Minor comments:
L12. A bit a weird sentence. Have not all species expanded at some point?
**> We have re-written the first two sentences of the abstract with this (and other comments in mind), so this sentence no longer exists.**

L15. Reproductive assurance instead of reproductive reassurance.

**> Fixed.**
